# Structural and functional properties of *Moringa stenopetala* seed meal protein isolates obtained from solvent-assisted protein extraction

**DOI:** 10.1186/s43014-026-00365-0

**Published:** 2026-04-01

**Authors:** Abisoye Dorcas Olukitibi, Anteneh Tesfaye Tefera, Debebe Worku Dadi, Mulualem Tamiru Kassa, Alphonsus Utioh, Rotimi Emmanuel Aluko

**Affiliations:** 1 Department of Food and Human Nutritional Sciences, University of Manitoba, Winnipeg, MB Canada; 2 BioTEI Inc., 200-135 Innovation Drive, Winnipeg, MB Canada; 3 ACU Food Technology Services Inc, Portage La Prairie, MB Canada; 4 Richardson Centre for Food Technology and Research, University of Manitoba, Winnipeg, MB Canada; 5 Food and Beverage Industry Research and Development Center, Addis Ababa, Ethiopia

**Keywords:** *Moringa stenopetala*, Protein isolate, Bitterness score, Emulsification, Foaming, Thermal properties, Protein digestibility

## Abstract

*Moringa stenopetala* (MS) is a stress-resilient, multipurpose African tree known for its valuable leaves as well as the protein and oil rich seeds. This study evaluated the effects of acetone (AMSPI), hexane (HMSPI), methanol (MMSPI), 100% ethanol (EMSPI), 70% ethanol (7EMSPI), 50% ethanol (5EMSPI), and water (WMSPI) pretreatments of MS seed meal on the physicochemical and functional properties of protein isolates obtained via alkaline isoelectric precipitation (ISO). These protein isolates were compared with the isolate produced from the untreated moringa seed meal (UMSPI). The protein content ranged from 85.34% for 5EMSPI to 96.95% for MMSPI. Protein isolates obtained from solvent-treated meals had higher contents of hydrophobic, positively charged, negatively charged, and sulfur containing amino acids than the UMSPI. Bitterness scores of the protein isolates obtained from solvent extracted MS meals ranged from 23.3 to 28.3 when compared to the 23.8 for the UMSPI while AMSPI and 7EMSPI had the highest scores of 28.3 ± 0.2 and 27.9 ± 0.1, respectively, as determined by electronic tongue. AMSPI demonstrated superior (*p* < 0.05) solubility (25–75% across pH 3–9), foaming capacity (~ 80%), and thermal stability (T_d_ = 88.77 °C) while MMSPI displayed the highest fluorescence intensity at pH 3. The UMSPI had the highest (*p* < 0.05) total phenolic content of 1.45 mg gallic acid equivalent (GAE)/g, while WMSPI (0.86 mg GAE/mg) had the lowest value. These findings highlight the significant influence of solvent extraction methods, with AMSPI and 7EMSPI emerging as optimal choices for food applications requiring high solubility, stability, and foaming properties.

**Graphical Abstract:**

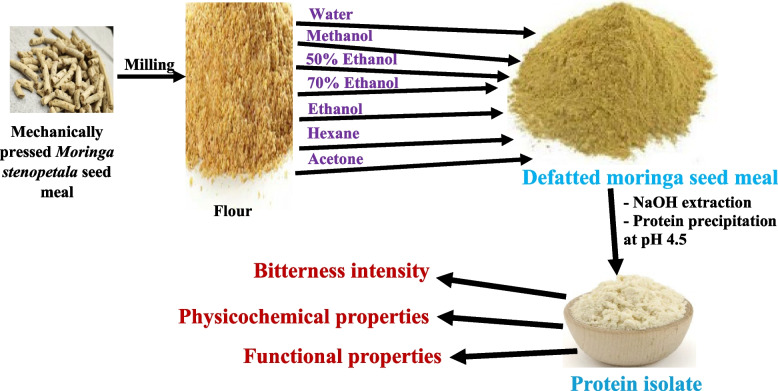

## Introduction

The rising popularity of plant-based proteins is driven by increased consumer awareness of their health benefits and the urgent need for sustainable food sources. Plant proteins serve as an excellent alternative to animal proteins, offering both nutritional value and unique functional characteristics (Sha & Xiong, 2020). These functional properties, such as emulsifying and foaming properties, are critical for determining their suitability in food and nutraceutical applications (Galanakis, 2021). However, the effectiveness of these proteins largely depends on their structural integrity, which is often altered during processing (Nunes & Tavares, 2019). Proteins generally remain in their native state, but processing steps during isolation can denature them, resulting in a loss of functionality. One significant step is solvent extraction to remove lipids, which plays a pivotal role in altering protein structure and performance. Various organic solvents, such as acetone, ethanol, and hexane, are often adopted to remove fat components. Hexane, a frequently utilized solvent in the food industry, presents concerns due to residual traces, which may pose health risks (Cravotto et al., 2022). Ethanol, on the other hand, is a safer alternative and has been successfully applied to defat soybeans (L’hocine et al., 2006), maize (Kwiatkowski & Cheryan, 2002), and *Quercus suber* fruits (Ferreira-Dias et al., 2003). In response to concerns about residual solvents, cold-press extraction has emerged as an alternative method to reduce solvent residues and minimize protein denaturation, as demonstrated in studies on *Hermetia illucens* larvae (Kim et al., 2022). Lipid removal is particularly crucial for oil-rich seeds, such as *Moringa stenopetala* (MS), which contain approximately 45% crude oil (Fang et al., 2023). However, isoelectric pH precipitation offers a proven approach to utilize the defatted meals for producing protein isolates that contain minimal levels of solvent residues.

MS serves as an important protein source, playing a crucial role in enhancing food security and improving nutrition (Horn et al., 2022). The seeds of MS contain approximately 36% protein and include all essential amino acids, making them a promising source of functional protein isolates (Melesse et al., 2011). With a balanced amino acid composition, MS protein isolate (MSPI) offers potential applications in human foods, providing an alternative to other plant or animal-based protein sources. The protein composition of MS seeds was compared to the FAO’s reference, hen’s egg protein, revealing a higher total amino acid content but lower essential amino acids (Olaofe et al., 2013). These findings highlight the potential of MS seeds for food applications, especially in underutilized plant-based food systems. The proteins derived from MS seeds are also highly digestible, making them an accessible source of amino acids (Seifu, 2015). This combination of digestibility and a complete amino acid profile enhance the value of MS in addressing protein deficiencies and combating malnutrition.

In the food industry, protein ingredients are valued for their structure, essential functionalities, and nutritional benefits. Previous research reports have indicated that the nutritional value and functionality of proteins are influenced by their structural conformation (Aryee et al., 2018; Wang et al., 2022). Reported studies have extensively explored the structure–function relationship in various plant proteins. For example, *Moringa oleifera*, a closely related species, has been well-researched for its protein functionality and potential application in food formulations (Aderinola et al., 2020; Bassogog et al., 2022). Research has shown that the seed proteins of *M. oleifera* exhibit excellent water and oil absorption capacities, which are critical for developing meat analogs, baked goods, and emulsified products like sausages and mayonnaise (Aderinola et al., 2020; Barbhai et al., 2024). However, similar research on MS remains limited. Despite its potential as a sustainable plant-based protein with bioactive and nutritional properties, the functional performance of MSPI have not been sufficiently explored, especially concerning the effect of solvent extraction methods on its structure and functionality.

In addition to the functional properties of proteins, sensory quality is a critical attribute, particularly when considering the five basic flavors that define human taste: bitterness, sourness, sweetness, saltiness, and umami (Ma et al., 2024). Bitterness is affected by several factors, such as the presence of specific compounds like polyphenols, alkaloids, and glucosinolates (Liu et al., 2024). In plant-based proteins, such as those derived from Moringa, bitter compounds tend to be concentrated in the protein isolate during extraction (Wong et al., 2023). The type of solvent used during protein extraction plays a crucial role in determining the bitterness of protein isolates. The method used for solvent-assisted extraction can either concentrate or reduce these bitter-tasting compounds, thereby impacting the overall sensory quality of the protein isolate. This consideration is especially relevant in product development, where the level of bitterness can significantly influence consumer acceptance. By carefully selecting the appropriate solvent, it may be possible to optimize the sensory profile of protein isolates, achieving a balance between functional properties and desirable taste characteristics.

While numerous studies have focused on the leaves of MS, limited attention has been given to the protein isolates derived from its seeds. Given the variability in solvent-assisted protein extraction techniques and the absence of comparative studies on MS protein isolates, it is crucial to examine how different solvents influence their functional properties. The current gap in research hinders our understanding of how processing steps, particularly defatting, affect the structural integrity and functionality of MS proteins. This study addressed this gap by offering a comprehensive evaluation of how different solvent-assisted protein extraction methods impact the functional properties of MSPI. While previous works have focused on *M. oleifera*, to the best of our knowledge, this is the first study to analyze the structural and functional properties of *M. stenopetala* seed protein isolates and to investigate the impact of various solvent-assisted protein extraction techniques on these properties. The findings will provide valuable insights for optimizing solvent-assisted protein extraction protocols to preserve and enhance the performance of MS proteins as suitable ingredients for food product formulations. Additionally, this work contributes to the development of sustainable plant-based protein alternatives, aligning with the growing demand for environmentally friendly and nutritionally beneficial food products.

## Materials and methods

### Materials

MS seed meal, produced through mechanical oil press was obtained from BioTEI Inc. (Winnipeg, MB, Canada) and stored at -20 °C. Double-distilled water (DDW) produced using a Millipore Milli-Q™ water purification system (Millipore Corp., Milford, MA, USA) was utilized throughout the research. Methanol, acetone, hexane, anhydrous ethanol, and all analytical grade chemicals used were obtained from Fisher Scientific (Oakville, ON, Canada) or Sigma Aldrich (St. Louis, MO, USA). Trypsin (lyophilized powder, 1,000–2,000 BAEE units/mg solid), chymotrypsin (Type II, lyophilized powder, ≥ 40 units/mg protein), peptidase (50–100 units/g solid) enzymes were purchased from Sigma Aldrich (St. Louis, MO, USA).

### Solvent extraction of MS meal

The MS meal was milled using a coffee grinder (PG-13658FA-CAN) to obtain a fine flour and then treated at room temperature (23–25 °C) with six different solvents (methanol, acetone, hexane, anhydrous ethanol, 50% ethanol, 70% ethanol) and water. A flour-to-solvent (or water) ratio of 1:10 (w/v) was maintained throughout the process. Each treatment was done in triplicate, stirred for 1 h, and then allowed to settle. The solvent (or water) phase was carefully decanted into storage containers, and the extraction process repeated for an additional 30 min. The solvent (or water) from the second extraction was combined with the initial extract. The solvent (or water) treated flour was spread thinly on an aluminum plate and left to dry overnight at 23–25 °C in a fume hood. The dried flour was weighed and stored in an airtight container at -20 °C, along with the untreated meal for comparison.

### Protein isolation using alkaline solubilization followed by isoelectric pH precipitation

MS seed meal or solvent (or water) treated flour was dispersed in 1 M NaOH at a 1:5 (w/v) ratio, stirred continuously for 1 h at 23–25 °C, and then centrifuged (8,000 × *g*, 1 h, 23–25 °C). The supernatant was collected, adjusted to pH 4.5 using 2.5 M HCl and stored at 4 °C for 12 h to facilitate protein precipitation. Following refrigeration, the acidified mixture was centrifuged again (8,000 × *g*, 1 h, 4 °C), and the precipitate freeze-dried to obtain the Moringa seed protein isolate (MSPI) followed by storage at -20 °C. The Moringa seed protein isolates (MSPIs) obtained from three separate extractions were combined and labelled as follows based on the solvent that was used to treat the original meal: MMSPI (methanol), AMSPI (acetone), HMSPI (hexane), EMSPI (anhydrous ethanol), 5EMSPI (50% ethanol), 7EMSPI (70% ethanol), and WMSPI (water).

### Proximate composition

The moisture, crude protein, dry matter, and ash contents of the protein isolates were determined using the methods specified by the Association of Official Analytical Chemists (Horwitz & Latimer, 2005). The crude fiber and fat contents were measured according to the procedures set by the American Oil Chemists' Society (Mehlenbacher 2010).

### Amino acid composition

Amino acid profile of the MSPIs was analyzed using the HPLC Pico-Tag system, following the method outlined by Bidlingmeyer et al. (1984). Regular amino acids were analyzed after sample hydrolysis with 6 M HCl for 24 h. Methionine and cysteine contents were determined following hydrolysis with performic acid (Gehrke et al., 1985), while alkaline hydrolysis was used for tryptophan (Landry & Delhaye, 1992). Briefly, amino acids in the hydrolyzed samples were derivatized by mixing 70 μL of Waters AccQ-tag buffer with 10 μL of the sample or standard and then 20 μL of AccQ-tag reagent, followed by incubation at 55 °C for 10 min. The regular amino acids were separated on the Nexera X2 UPLC system (Shimazdu Corp., Tokyo, Japan) attached to an AccQ-tag Ultra column. Samples were eluted using a gradient of Waters AccQTag buffers A and B over 17 min at 0.7 mL/min flow rate and 51 °C column temperature with UV detection at 260 nm. Determination of cysteine and methionine was achieved at 40 °C and 60 °C, respectively, using a 0.4 mL/min flow rate over 30 min with fluorescence detection at 266 nm excitation and 473 nm emission wavelengths. Analysis of tryptophan was performed after separation of the alkaline digest on a C18 column (Phenomenex, Torrance, CA, USA) using isocratic elution (1.0 mL/min flow rate at 28 °C and total run time of 34 min). The elution buffer was composed of 0.3% acetic acid, 0.05% 1,1,1-trichloro-2-methyl-2-propanol, and 5% methanol, and then adjusted to pH 5.0 with ethanolamine. Detection of tryptophan was achieved by measuring fluorescence intensity using 280 and 356 nm excitation and emission wavelengths, respectively.

### Intrinsic fluorescence

Intrinsic fluorescence of the samples was determined using the method of Agboola and Aluko (2009). Sample stock solutions containing 10 mg/mL were prepared using 0.1 M sodium phosphate buffer, pH 3, 5, 7, and 9. The mixture was then centrifuged, the supernatant collected, and its protein content quantified. The supernatant was diluted to 0.002% (w/v) using the same buffer solutions. The diluted protein samples were excited at a wavelength of 275 nm (tyrosine and tryptophan), and emission intensity was measured from 280 to 500 nm at 25 °C with the FP-6300 spectrofluorimeter (Jasco Inc., Tokyo, Japan). The fluorescence emission intensity (FI) of the buffer blanks was subtracted from the corresponding sample emission.

### Circular dichroism (CD)

Far-UV CD spectra were obtained following the method outlined by Agboola and Aluko (2009), with slight modifications using a J-815 spectropolarimeter (Jasco Corporation, Tokyo, Japan) at 23–25 °C. The far-UV CD spectrum was measured at a protein concentration of 2 mg/mL in 0.01 M phosphate buffer (pH 3.0–9.0) over a wavelength range of 190–240 nm, using a quartz cell with a 0.5 mm path length. CD spectra were averaged over three consecutive scans, with the corresponding buffer spectra automatically subtracted. The secondary structure of the protein fractions was analyzed from the far-UV data using the SELCON3 algorithm as described by Lobley et al. (2002) and Whitmore and Wallace (2004), available through the DichroWeb platform (http://dichroweb.cryst.bbk.ac.uk/html/home.shtml.).

### Total polyphenol content (TPC)

Samples were extracted with 80% methanol or 50% methanol or sequentially using 80% methanol, 50% methanol and 70% acetone followed by TPC determination by the Folin-Ciocalteu method as previously described (Agboola et al., 2010). A standard calibration curve was created using gallic acid concentrations ranging from 25 to 350 μg/mL in 50% (v/v) methanol. The samples were diluted to concentrations between 600 and 1400 μg/mL in 50% methanol. A 0.25 mL aliquot of either the gallic acid or protein isolate was combined with 0.25 mL of Folin-Ciocalteu reagent and incubated in the dark at 23–25 °C for 5 min. Then, 0.5 mL of a 20% (w/v) sodium carbonate solution and 4 mL of DDW were added to the mixture. After mixing thoroughly, the solution was incubated for 1 h in the dark. Absorbance of the resulting green color was measured at 725 nm using an Ultrospec UV–visible spectrophotometer (GE Healthcare, Montreal, PQ, Canada). The TPC was reported as the mean value from the 3 separate extractions and expressed as milligram gallic acid equivalents per gram (mg GAE/g) of sample.

### E-tongue analysis for bitterness intensity

The sample was weighed and dispersed in DDW at a concentration of 20 mg/mL followed by mixing at 23–25 °C for approximately 30 min to ensure complete extraction of the bitter compounds. After mixing, the solution was centrifuged at 5,000 × *g*, and the clear supernatant collected and filtered through 0.45 µm filter discs. For calibration and validation of the e-tongue, bitter standard solutions were prepared in reverse osmosis water at specific concentrations to serve as reference points for known bitterness levels. The standards included caffeine (0.05 mg/mL), acetaminophen (0.5 mg/mL), famotidine (0.02 mg/mL and 0.05 mg/mL), quinine (0.01 mg/mL and 0.04 mg/mL), loperamide hydrochloride (0.005 mg/mL), and denatonium benzoate (0.5 mg/mL). These compounds were used to construct a standard curve from which the bitterness score of the samples were obtained by linear regression.

### Surface hydrophobicity (S_o_)

The S_o_ of MSPIs was evaluated following the method described by Agboola and Aluko (2009), using 1-anilino-8-naphthalenesulfonate (ANS) as the fluorescent probe. The sample stock solution (1 mg/mL) was prepared in 10 mM phosphate buffer (pH 7.0) and stirred at 23–25 °C for 1 h, then centrifuged (7000 × *g*, 20 min, 23–25 °C). The supernatant was collected, analyzed for protein concentration and diluted with the phosphate buffer to give 30–250 µg/mL. A 40 µL aliquot of 8 mM ANS (prepared with the phosphate buffer) was added to 4.0 mL of each diluted sample solution. FI was measured at 390 and 470 nm excitation and emission wavelengths, respectively using the FP-6300 spectrofluorimeter (Jasco Inc., Tokyo, Japan). S_o_ was determined as the slope of a plot of FI versus protein concentration.

### Differential scanning calorimetry (DSC)

Thermal analysis was performed as previously described (Osemwota et al., 2021) using the TA Instruments Q100 differential scanning calorimeter (New Castle, DE, USA). Distilled water (10 mg) was placed into an aluminum pan, and 1 mg of the protein sample was added. The pan was then hermetically sealed and allowed to equilibrate at 23–25 °C for 2 h. The sealed pans were then heated at a rate of 10 °C/min, from 30 to 120 °C while an empty sealed pan served as a reference. The onset (T_o_) and denaturation (T_d_) temperatures as well as the enthalpy change (∆H) were determined using the Universal Analysis 2000 software (Version 4.5).

### Water and oil holding capacities

Water holding capacity (WHC) and oil holding capacity (OHC) were each determined using the method described by Malomo et al. (2014), which was slightly modified as follows. Samples were dispersed in water (WHC) or oil (OHC), vortexed for 1 min, allowed to stand for 30 min, centrifuged (5600 × *g*, 30 min, 23–25 °C) and the supernatant discarded. The precipitate was drained of excess water or oil for 15 min and weighed. WHC or OHC was determined as the sample weight gain and expressed as g of water (or oil) per g sample dry weight.

### In vitro protein digestibility (IVPD)

The IVPD was determined as previously described using an enzyme cocktail that consisted of 1.6 mg/mL trypsin, 3.1 mg/mL chymotrypsin, and 1.3 mg/mL peptidase (Hsu et al., 1977). The sample (6.25 mg/mL) was prepared in DDW, adjusted to 37 °C and pH 8.0 followed by addition of 1 mL of the enzyme mixture. The pH change after 10 min was determined, and the IVPD calculated using the following regression equation (Hsu et al., 1977).

1 $$IVPD \left(\%\right)=210.46-18.10Xf$$

where *Xf​* represents the final pH value of each sample after digestion for 10 min.

### Protein solubility (PS)

PS was assessed following the method previously described by Malomo et al. (2014), with slight modifications. Each sample was prepared as 10 mg/mL dispersions in 0.1 M acetate (pH 3 and 5), phosphate (pH 7), or Tris–HCl (pH 9) buffers. The mixtures were thoroughly vortexed, allowed to hydrate for 1 h, then centrifuged (5600 × *g*, 30 min, 23–25 °C) and the supernatant collected. Protein concentration in the supernatant was measured using the Lowry method (Markwell et al., 1978). Similarly, the total protein content of the MSPIs was determined by dissolving each of them in 0.1 M NaOH followed by determination of the concentration using the same method. Bovine serum albumin was used to prepare a calibration curve while absorbance readings were recorded at 660 nm and PS calculated as follows:

2 $$Protein\mathit\;solubility{\;(\%)}=\;\frac{\mathrm p\mathrm r\mathrm o\mathrm t\mathrm e\mathrm i\mathrm n\;\mathrm c\mathrm o\mathrm n\mathrm t\mathrm e\mathrm n\mathrm t\;\mathrm i\mathrm n\;\mathrm t\mathrm h\mathrm e\;\mathrm s\mathrm u\mathrm p\mathrm e\mathrm r\mathrm n\mathrm a\mathrm t\mathrm a\mathrm n\mathrm t}{\mathrm t\mathrm o\mathrm t\mathrm a\mathrm l\;\mathrm p\mathrm r\mathrm o\mathrm t\mathrm e\mathrm i\mathrm n\;\mathrm c\mathrm o\mathrm n\mathrm t\mathrm e\mathrm n\mathrm t\;\mathrm i\mathrm n\;\mathrm s\mathrm a\mathrm m\mathrm p\mathrm l\mathrm e}\times100$$

### Heat coagulability (HC)

HC was determined by dispersing the samples in buffer solutions at pH 3, 5, 7, and 9 as previously described by Malomo et al. (2014). The sample solution was then heated for 15 min in boiling water, followed by cooling to 23–25 °C. After cooling, the solutions were centrifuged (5600 × g, 30 min, 23–25 °C), the supernatant collected, and its protein content determined using the Lowry method (Markwell et al. 1978).

The HC calculation was done using this formula:

3 $$Heat\;coagulability{(\%)=}\frac{protein\mathit\;content\mathit\;of\mathit\;S\mathit1\mathit-protein\mathit\;content\mathit\;of\mathit\;S\mathit2\mathit\,}{protein\mathit\;content\mathit\;of\mathit\;S\mathit1}\times100$$

where S1 represents the total protein content of sample (before heating), and S2 represents the protein content of supernatant (after heating).

### Emulsion formation and stability

Oil-in-water emulsions were prepared using the method of Chao and Aluko (2018) with slight modifications. Samples (10, 15, and 20 mg protein/mL) were separately dispersed in 0.1 M acetate (pH 3 and 5), phosphate (pH 7), or Tris–HCl (pH 9) buffers. To create the emulsions, 1 mL of pure canola oil was mixed with 5 mL of sample dispersion. The mixture was homogenized for 1 min at 20,000 rpm using a Polytron PT 10–35 homogenizer (Kinematica AG, Lucerne, Switzerland), equipped with a 12-mm generator. The average oil droplet size (d₃,₂) was measured using a Mastersizer 3000 (Malvern Instruments Ltd., Malvern, UK), with Milli-Q water as the dispersing medium. Each emulsion sample was continuously sheared and mixed with approximately 100 mL of distilled water using the small-volume wet dispersion unit (Hydro 3000) connected to the instrument until the desired obscuration level was reached. The oil droplet size of each emulsion was measured automatically in triplicate, and the average value was recorded. To assess the emulsion's stability, the d₃,₂ was measured again 30 min after preparation. The emulsion stability was calculated using the following equation:

4 $$Emulsion\mathit\;stability\;{(\%)=}\frac{Oil\mathit\;droplet\mathit\;size\mathit\;at\mathit\;\mathit0\mathit\;min\mathit\;{\mathit(\mathit d}\mathit3\mathit,\mathit2\mathit)\,}{Oil\mathit\;droplet\mathit\;size\mathit\;after\mathit\;\mathit{30}\mathit\;min\mathit\;{\mathit(\mathit d}\mathit3\mathit,\mathit2\mathit)}\times100$$

### Foaming capacity (FC) and foam stability (FS)

Foam formation was carried out following the method described by Chao and Aluko (2018). Sample dispersions (10, 15, and 20 mg protein/mL) were prepared in acetate (pH 3 and 5), phosphate (pH 7), or Tris (pH 9) buffers. The dispersions were each homogenized at 20,000 rpm for 1 min using a 20 mm shaft on a Polytron PT 3100 homogenizer (Kinematica AG, Lucerne, Switzerland). The FC, defined as the ability of the continuous phase to incorporate air, was determined as the mean of three measurements using the following equation:

5 $${\mathrm F\mathrm o\mathrm a\mathrm m\mathrm i\mathrm n\mathrm g\;\mathrm c\mathrm a\mathrm p\mathrm a\mathrm c\mathrm i\mathrm t\mathrm y\;(\%)=}\frac{\mathrm V\mathrm o\mathrm l\mathrm u\mathrm m\mathrm e\;\mathrm a\mathrm f\mathrm t\mathrm e\mathrm r\;\mathrm h\mathrm o\mathrm m\mathrm o\mathrm g\mathrm e\mathrm n\mathrm i\mathrm z\mathrm a\mathrm t\mathrm i\mathrm o\mathrm n-\mathrm V\mathrm o\mathrm l\mathrm u\mathrm m\mathrm e\;\mathrm b\mathrm e\mathrm f\mathrm o\mathrm r\mathrm e\;\mathrm h\mathrm o\mathrm m\mathrm o\mathrm g\mathrm e\mathrm n\mathrm i\mathrm z\mathrm a\mathrm t\mathrm i\mathrm o\mathrm n}{\mathrm{Volume}\;\mathrm{before}\;\mathrm{homogenization}}\times100$$

FS was evaluated after 30 min at room temperature by measuring the volume of the remaining foam. This value was expressed as a percentage of the initial foam volume to assess the foam's capacity to retain its structure over time.

### Statistical analysis

Assays were conducted in duplicate or triplicate to determine mean values and standard deviations. Statistical data analysis was performed using one-way analysis of variance (ANOVA). Significant differences between mean values were assessed using Duncan's multiple range test (*p* < 0.05). All statistical analyses were carried out using the IBM SPSS statistical package (version 24, Armonk, NY, USA) and GraphPad Prism version 9.0 (GraphPad Software, San Diego, CA, USA).

## Results and discussion

### Effect of defatting on proximate composition of the MS seed meal

Table 1 presents the proximate composition of MS seed meal obtained using various defatting methods. Treatment of the meal with water resulted in the lowest moisture content, which suggests that the hydrophilic components with water-binding properties were more efficiently removed when compared to the use of solvents. Similar moisture reduction after aqueous processing of *M. oleifera* seeds was reported in an earlier study (Singh et al., 2011). However, solvent defatting with hexane and absolute ethanol produced the highest protein contents, which indicates the most efficient removal of non-protein materials. A comparable trend was observed by Haruna and Zannah (2023), who reported that crude protein levels in moringa seed meal significantly increased (up to ~ 55%) following oil extraction. Treatment of the meal with 50% ethanol led to the lowest protein content, an indication that some of the protein molecules were solubilized and removed by this solvent. This aligns with observations from comparative extraction studies showing that partial ethanol extraction can cause protein loss, whereas an optimized method such as the supercritical carbon dioxide-ethanol produced higher protein recovery (Senarathna & Malalgoda, 2024).

**Table 1:** Proximate composition of undefatted *Moringa stenopetala* (MS) meal (UMS) and meal defatted with acetone (AMS), 100% ethanol (EMS), hexane (HMS), methanol (MMS), 70% ethanol (7EMS), 50% ethanol (5EMS, and water (WMS)

Samples	Moisture (%)	Protein (%)	Fibre (%)	Fat (%)	Ash (%)	Non-fibre carbohydrates-by difference (%)
UMS	4.8 ± 0.1^e^	48.8 ± 0.1^e^	8.9 ± 0.3^b^	18.1 ± 0.0^c^	5.4 ± 0.2^c^	14.2 ± 0.0^c^
WMS	2.4 ± 0.4^f^	55.4 ± 0.1^c^	11.7 ± 1.8^a^	20.5 ± 1.5^b^	6.1 ± 0.6^ab^	3.8 ± 3.5^f^
HMS	5.7 ± 0.2^c^	57.4 ± 0.4^a^	8.3 ± 0.2^c^	1.4 ± 0.0^f^	6.6 ± 0.0^a^	20.5 ± 0.4^a^
AMS	5.2 ± 0.1^d^	55.6 ± 0.5^bc^	8.0 ± 0.0^c^	5.8 ± 1.2^e^	6.5 ± 0.3^a^	18.9 ± 0.5^b^
EMS	5.1 ± 0.2^d^	56.2 ± 0.3^b^	7.6 ± 1.5^c^	5.7 ± 1.0^e^	6.3 ± 0.2^a^	19.1 ± 0.8^ab^
5EMS	6.5 ± 0.0^b^	46.3 ± 0.3^f^	9.5 ± 1.3^b^	22.7 ± 0.5^a^	6.1 ± 0.2^ab^	8.9 ± 1.7^e^
7EMS	7.1 ± 0.5^a^	49.6 ± 1.1^de^	9.0 ± 1.0^b^	20.8 ± 1.1^b^	5.9 ± 0.2^b^	7.6 ± 0.7^e^
MMS	7.7 ± 0.9^a^	50.2 ± 0.9^d^	8.8 ± 0.9^b^	16.2 ± 1.9^d^	5.4 ± 0.2^c^	11.8 ± 0.9^d^

The data represent the mean values of duplicate determinations with their corresponding standard deviations

Different superscripts within the same column signify statistically significant differences among the mean values (*p* < 0.05)

As expected, water treatment was the least effective in removing lipids from the meal, and this was evident in the higher fat content, consistent with findings on moringa seed flour, where aqueous treatments left higher residual oil compared with solvent-extracted meals (Haruna & Zannah, 2023). In contrast, solvents like hexane, acetone, and absolute ethanol had the weakest ability to remove non-fibre carbohydrates, whereas water alone or water–ethanol mixtures improved carbohydrate removal, as evident in the lower values for the water-treated Moringa seed meal (WMS), 50% ethanol-treated M. *oringa* seed meal (5EMS) and 70% ethanol-treated Moringa seed meal (7EMS). This agrees with a previous study showing that aqueous or mixed extractions enhanced the reduction of soluble carbohydrates in defatted moringa seed flour (Singh et al., 2011). Fibre content was highest in the water-treated meal, supporting previous reports that aqueous defatting may retain insoluble fibrous residues (Haruna & Zannah, 2023). On the other hand, ash content generally increased after both water and solvent treatment, except for the methanol-treated meal, which had a similar value to the untreated meal. This observation is consistent with an earlier finding that defatting and extraction processes tend to concentrate mineral residues in moringa seed meals (Haruna & Zannah, 2023).

### Proximate composition of protein isolates

Table 2 presents the proximate composition of protein isolates derived from the defatted and undefatted meals. The data highlight differences in moisture, crude protein, fat, fiber, ash, and dry matter content among the samples, reflecting the impact of defatting and protein isolation process on their composition. Protein isolates obtained from meals treated with 70% ethanol (7EMSPI), methanol (MMSPI), and hexane (HMSPI) had significantly (*p* < 0.05) lower moisture contents when compared to the defatted meals. This reduction is expected because the protein isolation process involves extensive washing, centrifugation, and drying, which removes free water from the sample. The lower moisture content in the isolates contributes to their higher stability and longer shelf life when compared to the defatted meals. In contrast, the protein content increased significantly after protein isolation in comparison to the defatted meals. For example, MMSPI had a protein content of 96.95% when compared to the 50.2% for the methanol defatted meal (MMS). The increases occurred because the protein isolation process removes non-protein components such as fiber, fat, and carbohydrates. The results suggest that defatting did not have a negative effect on the isoelectric pH precipitation method with respect to the separation of proteins from non-protein components. The higher protein content of the protein isolates is reflected in the lower levels of the non-protein components, such as fibre and fat. The fat content was significantly reduced in the protein isolates (0.03–0.12%) when compared to the defatted meals (7.6–11.7%). However, the protein isolates had slightly higher ash contents (4.21–9.90%) than the defatted meals (5.4–6.6%). This is likely due to the formation of NaCl molecules during the isoelectric pH precipitation step when HCl was added to the alkaline protein extract.

**Table 2:** Proximate composition of solvent-assisted extracted *Moringa stenopetala* protein isolates

Samples	Moisture (%)	Protein (%)	Fibre (%)	Fat (%)	Ash (%)
UMSPI	1.48 ± 0.06^d^	85.34 ± 0.11^f^	0.04 ± 0.02^bc^	1.42 ± 0.09^c^	8.17 ± 0.21^c^
MMSPI	1.23 ± 0.01^de^	96.95 ± 0.02^a^	0.11 ± 0.02^a^	0.50 ± 0.15^d^	4.21 ± 0.01^f^
HMSPI	1.19 ± 0.03^de^	91.19 ± 0.12^c^	0.07 ± 0.01^b^	0.79 ± 0.08^d^	5.54 ± 0.03^e^
AMSPI	1.54 ± 0.02^d^	89.39 ± 0.14^d^	0.12 ± 0.01^a^	1.31 ± 0.07^c^	6.11 ± 0.00^d^
EMSPI	1.89 ± 0.05^c^	88.58 ± 0.20^e^	0.04 ± 0.01^bc^	2.02 ± 0.02^b^	6.29 ± 0.06^d^
7EMSPI	0.92 ± 0.02^e^	95.00 ± 0.13^b^	0.07 ± 0.01^bc^	1.17 ± 0.02^c^	5.58 ± 0.12^e^
5EMSPI	2.88 ± 0.31^b^	84.36 ± 0.07^g^	0.03 ± 0.00^c^	3.62 ± 0.21^a^	9.90 ± 0.01^a^
WMSPI	3.34 ± 0.01^a^	88.35 ± 0.03^e^	0.07 ± 0.01^bc^	3.81 ± 0.10^a^	8.57 ± 0.19^b^

The data represent the mean values of duplicate determinations with their corresponding standard deviations

Different superscripts within the same column signify statistically significant differences among the mean values (*p* < 0.05)

*UMSPI* undefatted MS meal protein isolate, *AMSPI* acetone defatted MS meal protein isolate, *EMSPI* 100% ethanol defatted MS meal protein isolate, *HMSPI* hexane defatted MS meal protein isolate, *MMSPI* methanol defatted MS meal protein isolate, *7EMSPI* 70% ethanol defatted MS meal protein isolate, *5EMSPI* 50% ethanol defatted MS meal protein isolate, *WMSPI* water defatted MS meal protein isolate

When compared to each other, the WMSPI exhibited the highest moisture content (3.34%), likely due to the use of water during the defatting process, which may arise from increased exposure of hydrophilic groups. In contrast, 7EMSPI had the lowest moisture content, attributed to the combined effect of water and 70% ethanol, which efficiently extracted both lipids and polar components without excessive structural disruption. A lower moisture content improves the stability and shelf life of protein isolates, reducing microbial activity and spoilage risk (Joshi et al., 2011). The moisture content in this study aligns with the recommended range of 3 – 4% for dried food products (Illingworth et al., 2024). MMSPI had the highest protein concentration, suggesting that methanol defatting effectively retains proteins. The protein content of MMSPI was significantly different (*p* < 0.05) from other isolates, including AMSPI and HMSPI, though these isolates also exhibited high values. 5EMSPI displayed the lowest protein content among the isolates. The higher protein content in MS seeds compared to Moringa leaves (27.3–30.2%) as reported by Mikore and Mulugeta (2017) demonstrates the nutritional superiority of the seeds. The protein content obtained in this work is comparable to the values reported for flaxseed (Kaushik et al., 2016) and lentil (Joshi et al., 2015) protein isolates.

Methanol and hexane defatting were particularly effective in reducing the fat content of the protein isolates, as shown by the lower fat concentrations in MMSPI and HMSPI, respectively. However, WMSPI had the highest fat content, which could be due to the poor fat removal in the meal as evident in the high fat content of the water-extracted meal (Table 1). Among the MSPIs produced from ethanol-treated meals, the 7EMSPI had the lowest fat content, suggesting that 70% ethanol treatment may have reduced protein-lipid interactions better than both absolute and 50% ethanol. The fat levels observed in this work are higher than those reported for pea (Cui et al., 2020) and lentil protein isolates (Osemwota et al., 2021). The fibre contents of the MSPIs were generally low, which indicates the efficiency of the isoelectric pH precipitation method in excluding fibre from the precipitated material during protein isolation. Ash contents in the MSPIs (4.21–9.90%) are superior to the 3.19–3.83% values that were reported for protein isolates isolated from lentil (Joshi et al., 2011) and other legumes such as soybeans, lentils, and chickpeas (Karaca et al., 2011).

### Amino acid composition

The amino acid composition is important because it influences nutritional quality, functional properties, and biological activity of protein isolates (Day et al., 2022). The essential amino acids (EAA) content was generally low for all the protein isolates (Table 3). The EAA profile of MSPI is similar to flaxseed protein isolate but lower than animal proteins such as the yellow mealworm larvae (44.93%) (Zhao et al., 2016) and hen’s egg proteins (43.6%) (Mishyna et al., 2019), while being higher than soy protein isolate (Kaushik et al., 2016). A high EAA content is particularly valuable for nutritional supplements aimed at supporting the health of low-birth-weight infants (Furuta et al., 2021). Consequently, MMSPI may be especially suitable for some infant foods. The lysine-to-arginine (Lys/Arg) ratio is a significant factor in assessing a protein's potential effects on cholesterol and cardiovascular health (Strauss et al., 2020). A lower Lys/Arg ratio is associated with reduced cholesterol levels and a reduced risk of atherosclerosis (Sanchez & Hubbard, 2018); however, human studies are required to confirm such health benefits. This benefit is attributed to arginine's positive effects on cardiovascular health, such as improved blood vessel function and reduced blood pressure, whereas a high lysine intake relative to arginine has been linked to increased cholesterol and atherogenic effects (Gambardella et al., 2020; Venkatesh et al., 2017). In this study, all MSPIs showed similar Lys/Arg ratios, indicating comparable effects on lipid metabolism. These values are significantly lower than those observed in other plant-based protein isolates. For instance, flaxseed protein isolate has a Lys/Arg ratio of 0.22 (Marambe et al., 2008), and soy protein isolate has a much higher ratio of 0.71 (Kaushik et al., 2016). Given the lower Lys/Arg ratios, MSPI may offer a health advantage in food formulations aimed at reducing lipid levels and atherogenic risks.

**Table 3:** Amino acid composition (%) of solvent-assisted extracted *Moringa stenopetala* protein isolates

Amino acids	EMSPI	MMSPI	HMSPI	AMSPI	UMSPI	7EMSPI	5EMSPI	WMSPI
His	1.64 ± 0.09^bc^	1.74 ± 0.04^bc^	1.60 ± 0.11^bc^	1.70 ± 0.02^bc^	1.53 ± 0.12^c^	1.68 ± 0.00^bc^	1.54 ± 0.04^bc^	1.81 ± 0.11^ab^
Ser	1.94 ± 0.01^cd^	2.15 ± 0.01^ab^	2.04 ± 0.06^bc^	1.91 ± 0.03^de^	1.85 ± 0.00^de^	2.06 ± 0.02^ab^	1.81 ± 0.05^f^	1.84 ± 0.01^ef^
Arg	9.50 ± 0.09^de^	10.76 ± 0.09^ab^	10.07 ± 0.40^bcd^	9.77 ± 0.15^cd^	9.14 ± 0.21^ef^	10.59 ± 0.20^ab^	8.80 ± 0.15^f^	10.37 ± 0.01^bc^
Gly	2.76 ± 0.00^cde^	2.98 ± 0.02^ab^	2.90 ± 0.10^bcd^	2.80 ± 0.02^bcd^	2.62 ± 0.06^ef^	2.91 ± 0.05^bc^	2.72 ± 0.06^de^	2.62 ± 0.04^ef^
Asx	4.36 ± 0.01^bd^	4.75 ± 0.01^ab^	4.57 ± 0.12^bc^	4.18 ± 0.09^de^	4.13 ± 0.02^de^	4.57 ± 0.00^bc^	4.24 ± 0.13^de^	4.06 ± 0.11^e^
Glx	15.07 ± 0.00^ef^	16.83 ± 0.12^a^	15.92 ± 0.52^bc^	15.12 ± 0.02^cde^	14.43 ± 0.19^ef^	16.80 ± 0.05^a^	14.51 ± 0.37^ef^	15.28 ± 0.03^cd^
Thr	1.87 ± 0.02^cd^	2.03 ± 0.01^ab^	1.94 ± 0.07^bc^	1.87 ± 0.01^cd^	1.74 ± 0.05^de^	1.97 ± 0.04^bc^	1.78 ± 0.03^de^	1.87 ± 0.01^cd^
Ala	2.84 ± 0.00^cde^	3.09 ± 0.22^ab^	3.00 ± 0.08^bc^	2.83 ± 0.03^cde^	2.71 ± 0.03^ef^	2.98 ± 0.03^cde^	2.68 ± 0.06 ^f^	2.83 ± 0.01^cde^
Pro	3.95 ± 0.06^c^	4.71 ± 0.53^ab^	4.25 ± 0.14^bc^	3.98 ± 0.00^c^	3.74 ± 0.07^c^	4.24 ± 0.06^bc^	3.67 ± 0.08^c^	3.84 ± 0.06^c^
Cys	1.00 ± 0.05^cd^	1.26 ± 0.05^a^	0.95 ± 0.06^d^	1.03 ± 0.01^cd^	0.81 ± 0.03^e^	1.30 ± 0.00^a^	1.09 ± 0.05^bc^	1.30 ± 0.04^a^
Lys	1.25 ± 0.04^bc^	1.34 ± 0.04^ab^	1.28 ± 0.04^bc^	1.20 ± 0.03^cd^	1.18 ± 0.02^cd^	1.22 ± 0.01^bc^	1.09 ± 0.01^d^	1.09 ± 0.05^d^
Tyr	1.47 ± 0.01^cd^	1.67 ± 0.01^ab^	1.59 ± 0.08^b^	1.52 ± 0.01^bc^	1.41 ± 0.04^d^	1.60 ± 0.06^b^	1.46 ± 0.03^cd^	1.47 ± 0.05^cd^
Met	1.43 ± 0.07^cd^	1.59 ± 0.01^ab^	1.41 ± 0.05^cde^	1.41 ± 0.04^cde^	1.25 ± 0.04^f^	1.48 ± 0.03^bc^	1.30 ± 0.03^ef^	1.37 ± 0.07^cde^
Val	3.43 ± 0.01^bc^	3.82 ± 0.02^ab^	3.65 ± 0.13^bc^	3.46 ± 0.02^bc^	3.27 ± 0.01^cd^	3.67 ± 0.06^bc^	3.28 ± 0.06^cd^	3.28 ± 0.03^cd^
Ile	2.59 ± 0.05^cd^	2.82 ± 0.04^ab^	2.76 ± 0.09^ab^	2.58 ± 0.02^cd^	2.48 ± 0.00^de^	2.71 ± 0.05^bc^	2.39 ± 0.06^ef^	2.57 ± 0.01^cd^
Leu	4.89 ± 0.03^ef^	5.42 ± 0.05^ab^	5.19 ± 0.19^bc^	4.96 ± 0.04^cde^	4.66 ± 0.08^fg^	5.22 ± 0.07^b^	4.63 ± 0.12^g^	5.00 ± 0.05^bc^
Phe	3.28 ± 0.04^cd^	3.60 ± 0.02^ab^	3.46 ± 0.13^b^	3.31 ± 0.08^cd^	3.15 ± 0.06^de^	3.40 ± 0.09^bc^	3.11 ± 0.07^de^	3.25 ± 0.06^cd^
Trp	0.92 ± 0.03^cd^	1.04 ± 0.00^a^	0.97 ± 0.00^bc^	0.95 ± 0.01^cd^	0.93 ± 0.00^cd^	0.93 ± 0.01^cd^	0.90 ± 0.03^d^	0.93 ± 0.03^cd^
AAA	5.68 ± 1.23^a^	6.31 ± 1.33^a^	6.01 ± 1.30^a^	5.78 ± 1.23^a^	5.59 ± 1.17^a^	5.92 ± 1.27^a^	5.46 ± 1.15^a^	5.65 ± 1.21^a^
BCAA	10.91 ± 1.16^a^	12.06 ± 1.31^a^	11.60 ± 1.22^a^	11.00 ± 1.2^a^	10.41 ± 1.10^a^	11.60 ± 1.27^a^	10.30 ± 1.13^a^	10.85 ± 1.25^a^
HAA	24.37 ± 1.36^a^	27.43 ± 1.52^a^	25.82 ± 1.47^a^	24.62 ± 1.37^a^	23.16 ± 1.31^a^	26.05 ± 1.42^a^	23.21 ± 1.25^a^	24.47 ± 1.32^a^
PCAA	12.39 ± 4.65^a^	13.84 ± 5.33^a^	12.95 ± 4.99^a^	12.67 ± 4.81^a^	11.85 ± 4.50^a^	13.49 ± 5.28^a^	11.43 ± 4.33^a^	13.27 ± 5.16^a^
NCAA	19.43 ± 7.57 ^a^	21.58 ± 8.54^a^	20.49 ± 8.03^a^	19.30 ± 7.74^a^	18.56 ± 7.28^a^	21.38 ± 8.65^a^	18.74 ± 7.26^a^	19.34 ± 7.93^a^
SCAA	2.43 ± 0.30^a^	2.85 ± 0.23^a^	2.36 ± 0.33^a^	2.44 ± 0.27^a^	2.06 ± 0.31^a^	2.78 ± 0.13^a^	2.39 ± 0.15^a^	2.67 ± 0.05^a^
EAA	21.30 ± 1.29^a^	23.4 ± 1.44^a^	22.26 ± 1.40^a^	21.44 ± 1.31^a^	20.19 ± 1.24^a^	22.28 ± 1.40^a^	20.02 ± 1.24^a^	21.17 ± 1.31^a^
NEAA	42.88 ± 4.61^a^	48.19 ± 5.16^a^	45.28 ± 4.88^a^	43.15 ± 4.66^a^	40.84 ± 4.44^a^	47.05 ± 5.16^a^	40.97 ± 4.39^a^	43.60 ± 4.78^a^

The data represent mean values ± standard deviation

*HAA* hydrophobic amino acids: Ala, Val, Ile, Leu, Tyr, Phe, Trp, Pro, Met, and Cys, *PCAA* positively charged amino acids: Arg, His, Lys; *NCAA* negatively charged amino acids: Asx – (asparagine + aspartic acid) and Glx (glutamine + glutamic acid), *AAA* aromatic amino acids: Phe, Trp, and Tyr, *SCAA* sulfur containing amino acids: Cys and Met, *BCAA* branched chain amino acids: Val, Leu, and Ile, *EAA* essential amino acids: Thr, Val, Met, Ile, Leu, Phe, His, Lys, and Trp, *NEAA* non-essential amino acids: Ala, Arg, Asx (asparagine + aspartic acid), Cys, Glx (glutamic acid + glutamine), Gly, Pro, Ser, and Tyr

*UMSPI* undefatted MS meal protein isolate, *AMSPI* acetone defatted MS meal protein isolate, *EMSPI* 100% ethanol defatted MS meal protein isolate, *HMSPI* hexane defatted MS meal protein isolate, *MMSPI* methanol defatted MS meal protein isolate, *7EMSPI* 70% ethanol defatted MS meal protein isolate, *5EMSPI* 50% ethanol defatted MS meal protein isolate, *WMSPI* water defatted MS meal protein isolate

The total non-essential amino acids (NEAA) content across all samples exceeded the EAA content, with NEAA levels ranging from 40.84% (UMSPI) to 48.19% (MMSPI). This aligns with reports that plant proteins generally have higher proportions of NEAAs, such as Glu, Asp, Ala, and Gly, compared to EAAs like Lys, Met, and Trp. This observation supports findings by Gorissen and Witard (2018), who indicated that plant-based proteins often have lower Met and Lys levels. Among the NEAAs, Arg (8.80–10.76%) and Glx (14.43–16.83%) were the most abundant. Glu plays a key role in nitrogen metabolism and serves as a precursor for gamma-aminobutyric acid, a neurotransmitter (Sulieman et al., 2024). NEAA levels in MSPI are similar to those found in *M. oleifera* leaves (Olaofe et al., 2013), lower than those of hen’s egg (54.7%) and cow’s milk (56.1%) (Mishyna et al., 2019), but higher than *Moringa oleifera* seed cake powder (Patil et al., 2022).

Branched-chain amino acids (BCAA) such as Leu, Ile, and Val, are essential for muscle repair and recovery. BCAA content ranged from 10.30% (5EMSPI) to 12.06% (MMSPI). In contrast, kidney bean vicilin reportedly has a higher BCAA content of about 18% (Mishyna et al., 2019). Hydrophobic amino acids (HAA) were highest in MMSPI at 27.43% and lowest in UMSPI at 23.16%, though the differences were not significant. HAA are commonly abundant in globular proteins, where they are often buried in the protein core or shielded by lipid layers (Bera et al., 2018). Solvents that interact with these proteins can influence the release or retention of HAA (Schwendeman et al., 2020). However, MMSPI had significantly the higher contents of Glx (glu + gln), and Trp than the other MSPIs in addition to comparably higher contents of Arg, Gly, Asx, Thr, Ala, Pro, Lys, Tyr, Met, Val, Ile, Leu, and Phe than most of the MSPIs. Methanol’s efficiency as a solvent may have better exposed these hydrophobic regions, enhancing extraction. These findings are comparable to those for chickpea protein isolate (Ghribi et al., 2015), though lower than lentil seed protein isolate (Osemwota et al., 2021). Positively charged amino acids (PCAA) like lysine and histidine are crucial for various biological functions. EMSPI, MMSPI, HMSPI, and 7EMSPI had the highest levels of lysine, while MMSPI, HMSPI, 7EMSPI, and WMSPI had the highest arginine contents. However, only the WMSPI had a higher level of His when compared to the UMSPI. Solvent polarity affects amino acid retention by influencing lipid dissolution. High-polarity solvents like methanol and ethanol can dissolve polar lipids, potentially preserving polar amino acids like arginine and glutamic acid. Similarly, aromatic amino acids (AAA), such as phenylalanine and tryptophan, are better retained in solvents that minimize protein denaturation; for instance, methanol effectively preserved a higher level of Trp.

The sulfur-containing amino acids (SCAA) content ranged from 2.06% in UMSPI to 2.85% in MMSPI. High SCAA levels enhance oxidative stability because of the strong antioxidant properties that help combat oxidative stress (Famuwagun et al., 2021). Although the relatively low SCAA levels are typical for plant proteins, Met and Cys are crucial for growth, cellular repair, and metabolism. Met is essential as a methyl donor in biochemical reactions, while Cys is a key component of the antioxidant glutathione (Kachungwa et al. 2022). Notably, MMSPI and 7EMSPI exhibited the highest Cys contents, values that are comparable to soybean protein isolate, higher than buckwheat protein isolate, but lower than rice protein isolate (Jin et al., 2021; Tang & Wang, 2010). Notably, the MSPIs obtained from the solvent extracted meal had significantly higher Cys levels than the isolate obtained from the untreated meal. In addition, except for 50% ethanol, other solvent extractions led to higher Met contents of the MSPIs. The results suggest that the use of water and solvents as pretreatment agents did not destroy the SCAAs, especially Met that is an essential amino acid.

### Intrinsic fluorescence

**Fig. 1:**
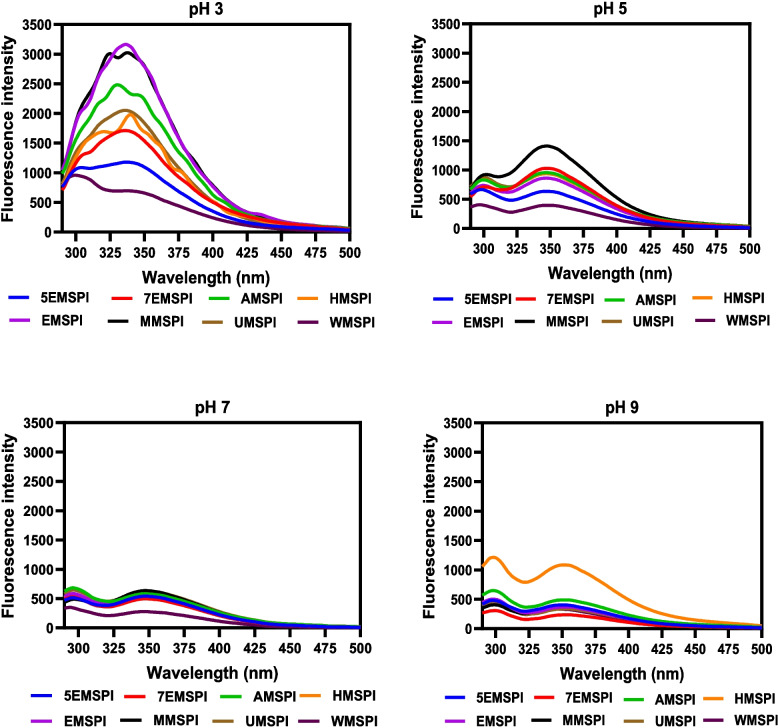
Intrinsic fluorescence of protein isolates obtained from undefatted *Moringa Stenopetala* meal (UMSPI) or meal defatted with; acetone (AMSPI), 100% ethanol (EMSPI), hexane (HMSPI), methanol (MMSPI), 70% ethanol (7EMSPI), 50% ethanol (5EMSPI), and water (WMSPI)

The intrinsic fluorescence analysis of MSPIs across varying pH levels (3, 5, 7, and 9) reveals structural shifts and changes in the exposure of aromatic amino acids, notably tryptophan and tyrosine, as shown in Fig. 1. FI, which depends on the environment of these aromatic residues, reflects tertiary structural alterations in the protein at different pH conditions (Wang et al., 2017). The spectra indicate a weak tyrosine peak at 303 nm and a more prominent tryptophan peak at 348 nm. The weak tyrosine signal, particularly at pH 3, may be due to its low quantum yield, contrasting with the strong emission of tryptophan. This suggests low surface exposure or minimal contribution, which may indicate either highly exposed tyrosine in a hydrophilic environment (Aderinola et al., 2020) or enhanced energy transfer from tyrosine to tryptophan due to strong protein–protein interactions (Malomo & Aluko, 2015). Similar to this study, previous reports have shown tyrosine emission maxima below 320 nm in *M. oleifera* seed protein isolate (Aderinola et al., 2020), and hemp seed protein isolate (Malomo & Aluko, 2015).

The tryptophan peak is consistently detectable across all pH levels, with the highest intensity observed at pH 3. This elevated intensity at pH 3 suggests reduced exposure of tryptophan residues to the hydrophilic environment and high degree of protein folding (Agboola & Aluko, 2009). As the protein transitions from a compact, folded state to a more exposed, and unfolded conformation, tryptophan emission shifts towards longer wavelengths with decreases in FI (Wei et al., 2019). At pH 5, near the isoelectric point of MS proteins, FI decreased due to protein aggregation and increased trapping of tryptophan in hydrophilic environments. Further decreases in tryptophan FI at pH 7 and 9 (except for HMSPI) indicate increased interactions with water. The lower FI observed at pH 5, 7, and 9 suggest a less compact protein structure, where aromatic and hydrophobic residues are far apart and engaged in more interactions with the hydrophilic environment (Du et al., 2022). Interestingly, as tryptophan FI decreased at pH 5, 7, and 9, tyrosine FI increased relative to its low level at pH 3, which indicates spatial separation from tryptophan residues (Jones & Farrens 2014). Notable differences in FI were observed among the isolates. WMSPI showed the lowest FI across most pH levels, except at pH 9. This indicates that water pretreatment of the MS meal resulted in a less compact structure of the isolated protein where aromatic and hydrophobic amino acids are exposed to the hydrophilic environment. This reduced compact conformation could explain the lower intensity, as fluorescence is generally weaker when aromatic residues like tryptophan are more exposed to the solvent environment. However, the increased FI of WMSPI at pH 9 indicates that there was enhanced interaction with the hydrophilic environment, which moved the aromatic groups into the protein core, hence reduced fluorescence quenching. Meanwhile, MMSPI exhibited the highest FI at most pH values, which suggest that methanol pretreatment of MS meal induced a compact, more folded protein structure that allowed aromatic residues to interact more readily with each other in the hydrophobic core of the protein and away from the hydrophilic environment. The results from this study are consistent with the observations reported by Yin et al. (2011) who showed that the FI of tryptophan and tyrosine residues vary significantly with changes in buffer pH from 3 to 9. This indicates that the microenvironment surrounding aromatic residues is highly sensitive to pH conditions. These findings further reinforce that protein structural conformations are affected by environmental factors, which influence folding behavior and the exposure of aromatic groups. Hence by determining FI at different pH levels, the ability of solvent pretreatment to modulate protein structure of oilseed meals when dispersed in different acid–base environments can be properly understood. Since food formulation occurs at different pH levels, the results provide a useful guide in determining the type of solvent to be used for specific end uses of the protein isolates.

### Secondary structure fractions

The far-UV CD spectrum is essential for understanding the secondary structure of proteins, providing insights into protein denaturation (Ruzza et al., 2021). In Table 4, the secondary structure of MSPI is predominantly unordered, followed by β-sheet and β-turn. This is different from the trend reported for oat bran proteins where β-sheet was predominant (Peng et al., 2026), suggesting that the processing of MSPI likely induced unfolding and structural rearrangements. The low α-helix content, consistently below 10% across most samples, suggests partial or complete denaturation. This is indicative of solvent interactions during defatting or pH-induced unfolding, with α-helices, generally being more stable in native proteins, lost during the isolation process. However, an exception is noted for MMGPI, which displays a significantly higher α-helix content at pH 3. A similar increase in α-helix content at low pH was reported by Jeong et al. (2021) for *Gryllus bimaculatus* protein concentrate, suggesting that specific extraction methods and conditions may help preserve helical structures to a degree. At pH 3 and 7, the protein isolate from hexane defatted meal had the lowest α-helix content. In contrast, the isolates from methanol and acetone defatted meals had lower α-helix contents at pH 5 and 9, respectively, which confirm the differential effects of solvents on protein conformation. Compared to previous findings, the α-helix content here is notably lower than the reported values for other plant proteins (Choi & Ma, 2007; Tang & Wang, 2010). The β-sheet content exhibits considerable variation depending on pH and defatting treatment, with its lowest value often seen at pH 7. Given that β-sheets contribute significantly to protein stability, this variability may reflect structural rearrangements where β-sheets decreased due to loss of inter molecular bonds. At pH 7, the reduced β-sheet content reflects conformational shifts that favor unordered structures and is correlated with the reduced solubility observed at this pH value.

**Table 4:** Secondary structure fractions of solvent-assisted extracted *Moringa stenopetala* protein isolates

pH	Structure	5EMGPI	7EMGPI	AMGPI	EMGPI	HMGPI	MMGPI	UMGPI	WMGPI
3	α-helix	9.20 ± 0.02^b^	3.95 ± 0.05^e^	4.80 ± 0.06^c^	3.85 ± 0.05^d^	2.85 ± 0.04^f^	23.05 ± 0.16^a^	3.30 ± 0.04^d^	4.85 ± 0.05^c^
	β-sheet	26.40 ± 0.10^f^	35.60 ± 0.16^c^	32.15 ± 0.14^e^	35.59 ± 0.16^c^	41.65 ± 0.19^a^	7.90 ± 0.05^g^	39.45 ± 0.18^b^	33.55 ± 0.15^d^
	β-Turns	15.90 ± 0.02^g^	19.0 ± 0.00^b^	17.55 ± 0.01^e^	18.25 ± 0.00^c^	19.55 ± 0.01^a^	17.10 ± 0.12^f^	19.60 ± 0.00^a^	17.75 ± 0.00^d^
	Unordered	52.0 ± 0.04^a^	41.45 ± 0.00^e^	45.65 ± 0.00^b^	41.90 ± 0.01^d^	35.85 ± 0.03^g^	52.0 ± 0.21^a^	37.65 ± 0.03^f^	43.80 ± 0.01^c^
5	α-helix	5.05 ± 0.05^e^	7.55 ± 0.02^b^	7.80 ± 0.04^a^	5.60 ± 0.05^d^	6.20 ± 0.05^c^	3.75 ± 0.05^h^	4.45 ± 0.06^g^	4.65 ± 0.05^f^
	β-sheet	29.45 ± 0.12^d^	14.65 ± 0.02^h^	20.05 ± 0.06^g^	26.00 ± 0.10^f^	27.85 ± 0.12^e^	36.15 ± 0.16^a^	30.10 ± 0.13^c^	33.70 ± 0.15^b^
	β-Turns	16.65 ± 0.01^e^	13.75 ± 0.03^h^	14.50 ± 0.02^g^	16.10 ± 0.02^f^	16.80 ± 0.02^d^	18.55 ± 0.00^a^	16.90 ± 0.01^c^	17.95 ± 0.00^b^
	Unordered	48.90 ± 0.02^e^	64.15 ± 0.15^a^	57.65 ± 0.08^b^	52.30 ± 0.05^c^	49.15 ± 0.06^d^	41.55 ± 0.02^h^	48.55 ± 0.02^f^	43.70 ± 0.02^g^
7	α-helix	6.20 ± 0.05^e^	9.10 ± 0.03^a^	5.90 ± 0.05^f^	8.05 ± 0.03^b^	4.95 ± 0.05^h^	7.05 ± 0.02^d^	7.35 ± 0.03^c^	5.55 ± 0.05^g^
	β-sheet	23.85 ± 0.09^c^	10.65 ± 0.01^h^	22.00 ± 0.07^d^	18.10 ± 0.05^e^	29.95 ± 0.13^a^	13.35 ± 0.01^g^	14.85 ± 0.02^f^	26.20 ± 0.10^b^
	β-Turns	15.55 ± 0.03^d^	14.85 ± 0.01^f^	15.40 ± 0.03^e^	13.95 ± 0.03^g^	16.95 ± 0.01^b^	18.00 ± 0.03^a^	13.95 ± 0.02^g^	15.75 ± 0.01^c^
	Unordered	54.45 ± 0.07^f^	65.35 ± 0.11^a^	56.70 ± 0.09^e^	59.95 ± 0.10^d^	48.15 ± 0.01^h^	61.55 ± 0.10^c^	63.90 ± 0.14^b^	52.45 ± 0.05^g^
9	α-helix	4.40 ± 0.05^f^	8.50 ± 0.03^a^	4.05 ± 0.05^g^	8.45 ± 0.04^a^	5.25 ± 0.04^d^	8.00 ± 0.04^b^	5.10 ± 0.05^e^	5.80 ± 0.05^c^
	β-sheet	35.65 ± 0.16^a^	22.00 ± 0.08^de^	35.75 ± 0.16^a^	22.40 ± 0.08^d^	34.00 ± 0.15^b^	21.70 ± 0.08^e^	30.15 ± 0.13^c^	29.80 ± 0.13^c^
	β-Turns	18.70 ± 0.01^b^	14.95 ± 0.02^f^	18.85 ± 0.00^a^	14.65 ± 0.02^g^	18.50 ± 0.01^c^	14.45 ± 0.03^h^	17.15 ± 0.01^d^	16.45 ± 0.01^e^
	Unordered	41.20 ± 0.03^f^	54.55 ± 0.08^b^	41.35 ± 0.00^f^	54.55 ± 0.07^b^	42.20 ± 0.02^e^	55.85 ± 0.08^a^	47.60 ± 0.02^d^	47.95 ± 0.01^c^

*UMSPI* undefatted MS meal protein isolate, *AMSPI* acetone defatted MS meal protein isolate, *EMSPI* 100% ethanol defatted MS meal protein isolate, *HMSPI* hexane defatted MS meal protein isolate, *MMSPI* methanol defatted MS meal protein isolate, *7EMSPI* 70% ethanol defatted MS meal protein isolate, *5EMSPI* 50% ethanol defatted MS meal protein isolate, *WMSPI* water defatted MS meal protein isolate

Within each row, values with different letters are significantly different (*p* < 0.05)

β-turns, which contribute to protein flexibility, remain relatively stable across pH levels and defatting methods, typically between 14 and 19%. Shapovalov et al. (2019) similarly reported that while β-turns are common, they do not dominate protein structure. High levels of unordered structure are observed in all samples, particularly in MMSPI and 7EMSPI. The unordered structure tends to increase at pH 7 and 9, likely due to further unfolding or denaturation as pH deviates from the isoelectric point. Mune Mune et al. (2017) found that increased unordered structures often correlate with higher surface hydrophobicity in protein isolates from grain legumes, as seen here in MMGPI and 7EMGPI, which also display high surface hydrophobicity likely due to greater exposure of hydrophobic residues in the unfolded structure. These results align with previous findings for similar plant proteins. Aderinola et al. (2020) and Osemwota et al. (2021) reported that unordered structures dominated the secondary structure of *M. oleifera* and lentil seed protein isolates, respectively. However, contrary to our findings, Illingworth et al. (2024) reported that α-helix and β-sheets predominated in quinoa protein isolates, accounting for about 58% of the structure, while D’Amico et al. (2017) also reported a predominance of β-sheets in amaranth protein isolates. This variability across different plant proteins may arise from their unique structural properties or extraction conditions. A comparison of the samples with UMSPI showed that water extraction of the MS meal produced the protein isolate (WMSPI) with the least changes in protein structure. In contrast, the protein isolate obtained from methanol extraction of the meal (MMSPI) had the most changes in structure, such as highest levels of α-helix at pH 3, β-sheet at pH 5, β-turns at pH 5 and 7, as well as unordered structure at pH 3 and 9. MMSPI also had the lowest contents of α-helix at pH 5, β-sheet at pH 3 and 9, β-turns at pH 9 and unordered at pH 5. Next to the MMSPI, protein isolates obtained from hexane and 70% ethanol treated meals also showed highly varied changes in secondary structure while ethanol defatting had less effects on protein structure when compared to the other organic solvents.

### Total polyphenol content (TPC)

Polyphenol content in foods is crucial for understanding their bioactivity, functionality, and potential health benefits (Rana et al., 2022). Monitoring these levels helps optimize formulations without compromising bioactivity (Gunasekaran et al., 2014). TPC of the MSPIs varied significantly based on the defatting methods used, as shown in Table 5. The WMSPI recorded lower TPC value than the protein isolate from the undefatted meal (UMSPI). Therefore, while solvent defatting reduced the TPC value, only the water extraction led to a significantly lower level when compared to the undefatted meal (UMSPI). The results suggest better solubilization of hydrophilic polyphenols, resulting in their higher removal during water-based defatting. This aligns with Jones and Farrens (2014), who reported that polyphenols from broccoli tissue were lost primarily through leaching into water, though the extent of loss depended on water volume. The TPC values obtained in this study are lower than those reported for *M. oleifera* seed extracts (Vyas et al., 2015). However, the results align more closely with values reported for pumpkin seed, rice, and pea protein isolates, though a higher TPC was recorded for hemp seed protein isolates (Sawicki et al., 2024). Apart from water extraction (WMSPI), which had significantly lower TPC than the original MS meal (UMSPI), solvent polarity did not correlate with TPC content.

**Table 5:** Total polyphenol content (TPC), bitterness score (BS), surface hydrophobicity (SH), differential scanning calorimetry (DSC), water holding capacity (WHC), oil holding capacity (OHC), and in vitro protein digestibility (IVPD) of solvent-assisted extracted *Moringa stenopetala* (MS) protein isolates

Sample		UMSPI	AMSPI	HMSPI	EMSPI	MMSPI	WMSPI	7EMSPI	5EMSPI
TPC (mg GAE/g)		1.45 ± 0.07^a^	1.17 ± 0.18^ab^	1.31 ± 0.06^ab^	1.35 ± 0.14^ab^	1.14 ± 0.22^ab^	0.86 ± 0.38^b^	0.98 ± 0.09^ab^	1.11 ± 0.21^ab^
BS		23.8 ± 0.1^f^	28.3 ± 0.2^a^	23.6 ± 0.1^f^	26.8 ± 0.1^c^	23.3 ± 0.1^fg^	24.2 ± 0.1^e^	27.9 ± 0.1^b^	26.7 ± 0.1^cd^
SH		13.6 ± 1.1^cd^	22.9 ± 0.1^b^	20.1 ± 2.1^b^	18.3 ± 0.2^bc^	32.7 ± 1.9^a^	12.4 ± 1.1^d^	23.6 ± 0.5^b^	6.1 ± 0.4^e^
DSC	T_o_ (^o^C)	85.1 ± 0.2^g^	85.0 ± 0.2^h^	85.5 ± 0.3^c^	85.2 ± 0.4^f^	85.3 ± 0.1^d^	85.9 ± 0.3^a^	85.2 ± 0.0^e^	85.6 ± 0.3^b^
	T_d_ (^o^C)	88.8 ± 0.1^c^	88.8 ± 0.2^c^	89.2 ± 0.4^b^	88.7 ± 0.0^c^	88.7 ± 0.0^c^	89.2 ± 0.5^b^	88.8 ± 0.0^c^	89.7 ± 0.5^a^
	∆H (J/g)	0.2 ± 0.0^b^	0.3 ± 0.0^a^	0.2 ± 0.0^b^	0.2 ± 0.0^b^	0.2 ± 0.1^b^	0.1 ± 0.0^c^	0.2 ± 0.0^b^	0.2 ± 0.0^b^
WHC (g/g)		2.2 ± 0.1^d^	2.3 ± 0.1^cd^	2.5 ± 0.1^b^	2.3 ± 0.2^cd^	2.2 ± 0.1^cd^	2.5 ± 0.1^b^	2.3 ± 0.0^c^	2.8 ± 0.1^a^
OHC (g/g)		3.2 ± 0.2^d^	3.7 ± 0.1^ab^	3.6 ± 0.2^bc^	3.0 ± 0.0^d^	3.0 ± 0.2^d^	3.5 ± 0.0^c^	4.0 ± 0.4^a^	3.8 ± 0.2^ab^
IVPD (%)		76.5 ± 0.4^ef^	78.7 ± 0.2^c^	77.0 ± 0.5^e^	79.0 ± 0.6^c^	80.1 ± 0.5^b^	77.2 ± 0.5^de^	81.3 ± 0.3^a^	78.0 ± 0.9^d^

T_o_ (onset temperature), T_d_ (maximum temperature), ∆H (enthalpy change)

The data represent the mean values of duplicate readings with their corresponding standard deviations

Different superscripts within the same row signify statistically significant differences among the samples (*p* < 0.05)

*UMSPI* undefatted MS meal protein isolate, *AMSPI* acetone defatted MS meal protein isolate, *EMSPI* 100% ethanol defatted MS meal protein isolate, *HMSPI* hexane defatted MS meal protein isolate, *MMSPI* methanol defatted MS meal protein isolate, *7EMSPI* 70% ethanol defatted MS meal protein isolate, *5EMSPI* 50% ethanol defatted MS meal protein isolate, *WMSPI* water defatted MS meal protein isolate

### Bitterness intensity

Bitterness is a critical sensory attribute that can significantly influence the acceptability of food products, especially when protein isolates are used in formulations. Compounds such as saponins, alkaloids, glucosinolates, and polyphenols are primarily responsible for the bitterness in Moringa seeds (Valdés-Rodríguez, 2023). However, it was suggested that glucosinolates are the primary contributors to Moringa's bitter taste (Kashyap et al., 2022). The protein isolate from undefatted MS seed meal exhibited a similar bitterness score to the protein isolates from methanol and hexane defatted meals (Table 5). In contrast, the bitterness scores of the MSPIs obtained from ethanol, acetone and water treatments increased significantly. These bitterness levels are higher than the 5.1 score that was reported for a soybean protein hydrolysate (Tong et al., 2020). The increased bitterness scores for some of the MSPIs suggest that the bitter compounds were bound to protein molecules and could not be efficiently removed by water, acetone, and methanol, hence they precipitated along with proteins and became enriched in the protein isolates. The relatively elevated bitterness score of the AMSPI could also be due to acetone’s high efficiency in removing lipids and non-polar compounds, which may have increased the levels of hydrophilic bitter compounds such as glucosinolates. There was no correlation between the bitterness score and TPC, which indicates that other bitter compounds, especially the glucosinolates are major determinants of the bitterness property as previously suggested (Kashyap et al., 2022). Overall, the results indicate that the defatting method intensified the concentration of bitter-tasting compounds in the MSPIs except for hexane and methanol. Moreover, the bitterness scores obtained for all the MSPIs exceed the 16.5 threshold, which is the tolerable limit indicated for bitter compounds (Rachid et al., 2010). However, the MSPIs will be used as food ingredients, and therefore, the bitterness scores of formulated products will be lower because of dilution effects.

### Surface hydrophobicity (S_o_)

S_o_ reflects the extent to which hydrophobic amino acid residues are exposed on the protein’s surface (Zhu et al., 2016). This property provides key insights into functional characteristics such as solubility, emulsification, and foaming capacity (Yan et al., 2021). Protein unfolding exposes hydrophobic residues that were previously buried within the protein’s core (Bassogog et al., 2022). The MSPIs from defatted meals showed significant (*p* < 0.05) variations in S_o_, indicating that the solvent type influences the exposure of hydrophobic residues during protein isolation (Table 5). MMSPI exhibited the highest S_o_, suggesting that methanol promotes greater unfolding and exposure of more hydrophobic residues of the proteins. In contrast, 5EMSPI had the lowest S_o_, implying better preservation of native protein structure with limited exposure of hydrophobic groups. Similarly, WMSPI showed relatively low S_o_, comparable to the isolate obtained from the undefatted meal (UMSPI). This suggests that water defatting causes less structural disruption than organic solvents, resulting in fewer exposed hydrophobic residues. On the other hand, 7EMSPI, AMSPI, HMSPI, and EMSPI recorded moderate S_o_ but still higher than the UMSPI, likely reflecting a mild denaturing effect that partially unfolds the protein, exposing some hydrophobic regions while retaining structural integrity. The S_o_ values observed in this study are lower than those reported for *M. oleifera* seed protein isolates, which recorded 50.38 (Aderinola et al., 2020). The variation in hydrophobicity across treatments underscores the role of defatting and solvent choice in modulating protein conformation and the extent of hydrophobic residue exposure. Higher S_o_ generally enhances protein–protein interactions, which can influence emulsifying and foaming properties (Chao et al., 2018). These findings are consistent with a previous report, which suggested that organic solvents can alter protein structures to either increase or decrease hydrophobic interactions, depending on the solvent's polarity and interactions with the protein matrix (Sanchez & Hubbard, 2018).

### Differential scanning calorimetry (DSC)

In this study, T_o_ and T_d_ values, which represent the onset and maximum temperature of protein denaturation, respectively were similar for all the samples, which indicate that the solvent type used for defatting the meal did not have significant effect on the strength of non-covalent bonds that held the protein molecules together (Table 5). However, most of the MSPIs had slightly higher T_o_, suggesting that these proteins showed more resistance to initial thermal denaturation. Therefore, it is likely that solvent pretreatment may have increased stability of the proteins, thereby mitigating thermal stress (Bilal et al., 2021). The T_o_ values obtained in this work surpass the 66.50 °C reported for pea protein concentrate (Asen & Aluko, 2022) but align closely with values reported for lentil protein isolates (Osemwota et al., 2021). The T_d_ denotes the peak temperature of denaturation and was also similar across the samples, except the slightly higher value for 5EMSPI, suggesting a more stable protein conformation. The T_d_ values are lower than the 94.30–95.05 °C reported for hemp seed protein isolates (Fang et al., 2023), underscoring variability in thermal tolerance across protein type. ΔH reveals the energy necessary for denaturation, directly correlating with structural stability (Seelig & Seelig, 2023). The AMSPI with the highest ΔH has the tightest protein conformation while the WMSPI had the lowest value, indicating a looser structural arrangement when compared to the other MSPIs. The ΔH values obtained in this work are lower than the 11.4 J/g that was reported for buckwheat globulin (Tang & Wang, 2010) and 75.93 J/g for chickpea protein isolate (Ghribi et al., 2015) but some of them are slightly higher than the 0.16 J/g for lentil protein isolates (Miranda et al., 2022).

### Water and oil holding capacity

The oil-holding capacity (OHC) and water-holding capacity (WHC) are critical functional properties of protein isolates, reflecting their ability to retain oils and water, respectively, in food systems (Zakki et al., 2024). These properties are essential for applications like emulsification, texture enhancement, and moisture retention in various food formulations (Ma et al., 2022). Table 5 shows that the highest WHC was observed in 5EMSPI, suggesting that the moderate ethanol concentration pretreatment of the MS meal enhanced water retention by favourably altering the protein structure. This could also be linked to its solubility, since samples with lower protein solubility exhibit higher WHC (Rezaei et al., 2022). HMSPI and WMSPI also showed relatively high WHC, while UMSPI and MMSPI exhibited the lowest WHC. The results suggest that defatting of the MS meal improved WHC of isolated proteins by restructuring the protein and exposing hydrophilic regions that enhance water-binding capacity (Nahimana et al., 2023). Therefore, HMSPI and WMSPI could be useful ingredients to formulate high moisture foods such as cakes, sausages, and soft energy (granola) bars and cookies. In contrast, UMSPI and MMSPI with poor WHC can be used to formulate dry food products such as hard energy (granola) bars and cookies, as well as protein-fortified puffed snacks.

Most of the MSPIs isolated from pretreated meals also had higher OHC than the UMSPI, suggesting that solvent defatting (especially with acetone, hexane, aqueous ethanol and water) may have partially changed the protein structure to expose hydrophobic groups, which contributed to enhanced affinity for oil (Table 5). This aligns with a previous report suggesting that increased exposure of hydrophobic amino acid residues tends to boost OHC (Aderinola et al., 2020). In contrast, defatting with absolute ethanol and methanol led to reduced OHC of the MSPIs, which suggests modification of the protein surface to expose less hydrophobic groups when compared to the other protein isolates. However, despite having only the second-highest hydrophobic amino acid content, 7EMSPI exhibited the highest OHC, indicating that factors beyond hydrophobicity such as structural conformation and physical characteristics like bulk density might also contribute. For example, Joshi et al. (2015) noted that higher bulk density can improve OHC by increasing oil absorption sites. OHC plays a vital role in new product development, enhancing flavor retention and slowing lipid oxidation, which delays rancidity (Zhang et al., 2024). High OHC also stabilizes fat-rich foods, extending shelf life and improving product quality (Aderinola et al., 2020). Notably, the OHC values for MSPI are lower than those reported for pea protein isolates (Asen & Aluko, 2022). The high OHC of 7EMSPI makes it a suitable ingredient to formulate lipid-rich foods such as mayonnaise, hamburgers, hot dogs, and sausages.

### In vitro protein digestibility

Table 5 presents the IVPD of the MSPIs, which reflects how easily the proteins can be broken down during digestion, an essential factor in evaluating their bioavailability and overall nutritional quality. The IVPD ranged from 76.52% to 81.32%, aligning with previous findings for roasted, debittered *Moringa peregrina* press cake (Sardabi et al., 2022), green lentil protein concentrate (Barbana & Boye, 2013), and pea protein concentrate (Çabuk et al., 2018). However, these values are lower than those reported for Kariya (Adiamo et al., 2016) and black and yellow quinoa (Sánchez-Reséndiz et al., 2019) protein isolates. Among the samples, 7EMSPI exhibited the highest digestibility, while UMSPI had the lowest. This suggests that pretreatment of the MS meal with solvents or water enhanced digestibility of subsequently isolated proteins, which may be due to removal of compounds that could interfere with protease activities. The variation in digestibility across samples indicates that the choice of defatting solvent plays a critical role. For instance, the reduced digestibility of HMSPI could suggest hexane-induced partial protein denaturation and aggregation while the high fat contents in WMSPI and 5MSPI may have contributed to the lower IVPD values. In contrast, the higher IVPD of 7EMSPI indicate a looser structure that provides better access to the proteases. High digestibility is advantageous for formulating protein supplements or functional foods aimed at enhancing protein intake and absorption, particularly for consumers requiring easily digestible plant-based proteins (Kuesten & Hu, 2020).

### Protein solubility (PS)

**Fig. 2:**
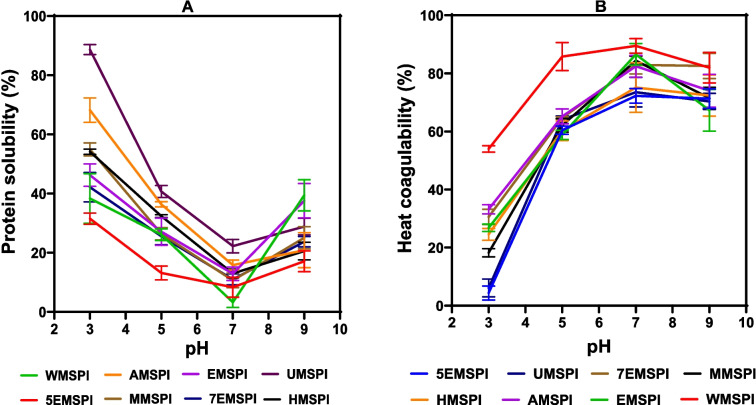
Protein solubility (**A**) and heat coagulability (**B**) of protein isolates obtained from undefatted *Moringa Stenopetala *meal (UMSPI) or meal defatted with; acetone (AMSPI), 100% ethanol (EMSPI), hexane (HMSPI), methanol (MMSPI), 70% ethanol (7EMSPI), 50% ethanol (5EMSPI), and water (WMSPI). The data represent the mean values of duplicate readings with their corresponding standard deviations

PS is a critical factor influencing the functional, sensory, and nutritional qualities of food products (Day et al., 2022). It affects essential aspects such as incorporation ease, stability, texture, and shelf life in food formulations (Hosseini & Jafari, 2020). Consequently, understanding the solubility behavior of food proteins is vital for optimizing product performance. Figure 2A illustrates the PS curve of MSPIs across a pH range of 3, 5, 7, and 9. The curve exhibits a U-shaped pattern, indicating that PS was lowest at pH 7. The decreases in PS at pH 5 to 7 suggest proximity to the isoelectric point, where the MS proteins carry minimal net charge and tend to aggregate, leading to reduced solubility. At pH 3 and 9, the proteins acquire net positive and negative charges, respectively, which increase electrostatic repulsion among the protein molecules to minimize aggregation and produce enhanced PS (Mundi & Aluko, 2012). However, the minimal PS at pH 7 is contrary to the pH 4–5 reported for other plant protein isolates from rice bran (Abd Rahim et al., 2024), and pea seed (Othmeni et al., 2024) but was positively correlated with the reduction in β-sheet (Table 4). This variation indicates that the MS meal contained more hydrophilic proteins than the typical plant proteins. It is also possible that there was more protein aggregation at pH 7 than pH 5 as previous research has shown that protein aggregates can form at neutral pH due to hydrophobic interactions, even if the isoelectric point is at a different pH (Vetri et al., 2007). The higher solubility observed at pH 3 indicates that the MS proteins possess a strong positive charge at this acidic pH, minimizing aggregation and maintaining good dispersion in solution. This finding aligns with studies on kidney bean albumin and buckwheat protein isolate, which also exhibited higher solubility at pH 3.

Among the MSPIs, UMSPI exhibited the highest PS across all pH levels, particularly at pH 3. This is likely due to the retention of the proteins' natural conformation, which prevented excessive unfolding and aggregation that occur during defatting (Levental & Lyman, 2023). The preservation of structural integrity allowed isolated proteins from the undefatted meal (UMSPI) to maintain higher PS over a broader pH range when compared to proteins isolated from the defatted meals. In contrast, 5EMSPI showed the lowest solubility across all pH levels except at pH 7, while AMSPI demonstrated relatively higher PS at most pH levels except pH 9. The results suggest that acetone effectively removed non-polar compounds like lipids and free fatty acids without significantly denaturing the proteins (Abhari & Khaneghah, 2020). This selective removal of non-polar compounds enhanced exposure of hydrophilic groups on the protein surface, which promoted interactions with water and improving PS.

### Heat coagulability (HC)

HC reflects the degree of protein aggregation upon heating, which is influenced by pH, protein structure, and pre-treatment methods. At pH 3, all samples showed minimal coagulability but there were sharp increases at pH 5 and 7 (Fig. 2B). At pH 9, HC decreased slightly, indicating reduced aggregation under alkaline conditions. This outcome contrasts with the findings of Osemwota et al. (2021) for lentil protein isolate, which reported a more pronounced decline in HC at alkaline pH. WMSPI consistently exhibited the highest HC across all pH levels, reaching a peak of 90% at pH 7, indicating rapid aggregation upon heating. In comparison, both HMSPI and UMSPI show lower HC across the pH range, reflecting weaker heat-induced aggregation. The HC curve inversely correlates with the PS curve. This agrees with previous research reports, which showed that reduced solubility during heating is associated with enhanced protein aggregation caused by molecular unfolding (Chen et al., 2018). Similarly, Grossman & McClements (2023) confirmed that lower solubility promotes heat-induced aggregation. The low HC of 5EMSPI and UMSPI at pH 3 indicate their potential use as ingredients to formulate food products that require pasteurization-resistant proteins.

### Emulsion formation and stability

**Fig. 3:**
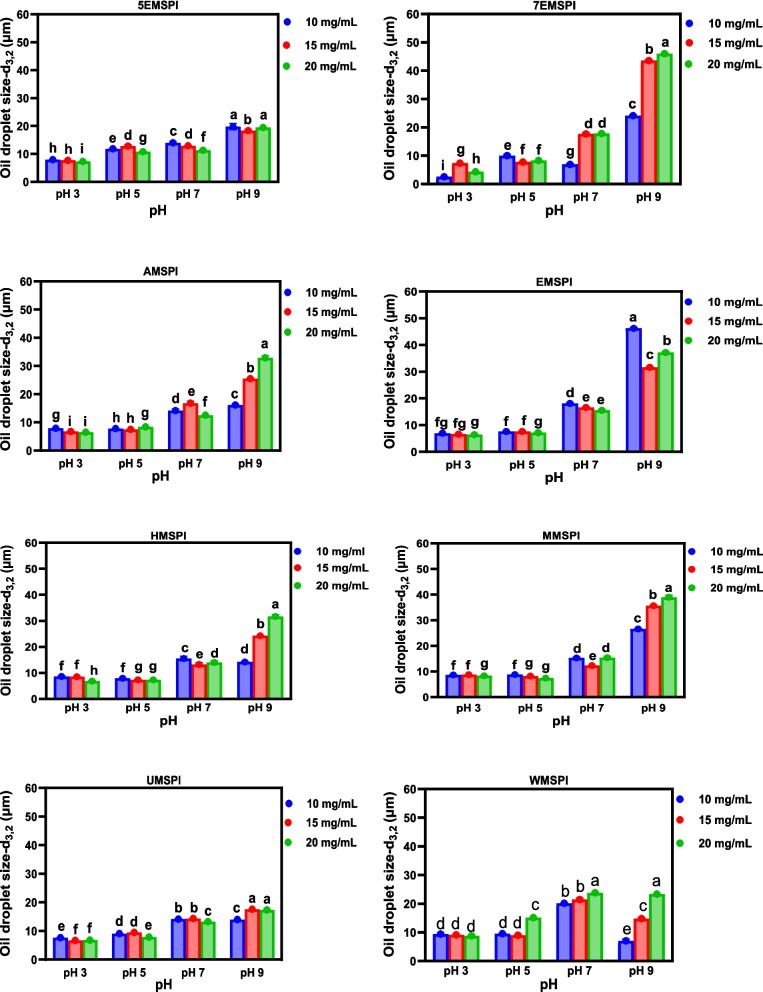
Oil droplet size of emulsions formed by protein isolates obtained from undefatted *Moringa Stenopetala* meal (UMGPI) or meal defatted with; acetone (AMGPI), 100% ethanol (EMGPI), hexane (HMGPI), methanol (MMGPI), 70% ethanol (7EMGPI), 50% ethanol (5EMGPI), and water (WMGPI). The data represent the mean values of duplicate readings with their corresponding standard deviations

Figure 3 presents the oil droplet size (d_3,2_) of emulsions formed with the MSPI at different protein concentrations (10, 15, and 20 mg/mL) and pH values (3, 5, 7, and 9). During emulsification, proteins interact with the oil–water interface, where they adsorb and create viscoelastic films that surround the oil droplets (Zhou et al., 2021). The emulsifying properties of MSPI were strongly pH-dependent, aligning with prior studies showing that emulsion capacity is influenced by pH, which affects the hydrophilic-hydrophobic balance (Illingworth et al., 2024). At pH 7 and 9, larger oil droplets were observed, indicating reduced emulsifying efficiency under alkaline conditions. In contrast, smaller droplets were formed at pH 3 and 5, suggesting better emulsifying performance in acidic environments. These results align with the findings from Osemwota et al. (2021), who reported similar trends for lentil protein isolates prepared using NaCl membrane methods. The effect of protein concentration was minimal at pH 3 and 5 but more pronounced at pH 7 and 9, where higher concentrations resulted in larger droplets, which suggests that increased electrostatic repulsions between the proteins prevent adequate formation of interfacial membranes needed for emulsification of the oil droplets. The performance of MSPI varied by type, with the UMSPI, WMSPI, and 5EMSPI consistently forming smaller droplets across all pH levels, including pH 7 and 9, indicating superior emulsifying properties. However, the 7EMSPI produced emulsions smaller emulsified oil droplets at pH 3, especially with the 10 mg/mL protein concentration. Conversely, EMSPI, 7EMSPI, and MMSPI produced larger droplets, particularly at pH 9. This suggests that, despite the high hydrophobic amino acid content in 7EMSPI and MMSPI, which enhanced emulsifying properties by reducing surface tension (Zhan et al., 2022), the use of methanol and high concentrations of ethanol during defatting led to structural changes in the proteins that increased exposure of hydrophilic groups at pH 9, thus reducing the formation of interfacial membranes. Generally, the trend in emulsion formation ability was positively related to the intrinsic FI, which suggests the proteins had a more ordered conformation at pH 3, but became disorganized as pH increased to 9. These findings have practical relevance for emulsified food products such as sauces, dressings, and nutraceutical beverages. The pH-dependent emulsifying behavior suggests that these isolates could perform better in acidic systems like yogurt, fruit beverages, and acidic sauces, where smaller droplet sizes and enhanced emulsifying properties are preferred. The oil droplet sizes reported here are smaller (indicating better emulsions) than the 18–85 µm reported for canola protein isolates (Tan et al., 2014) and 22–100 µm for pea protein isolates (Chao & Aluko, 2018). These differences underscore the importance of raw material selection in determining protein functionality.

**Fig. 4:**
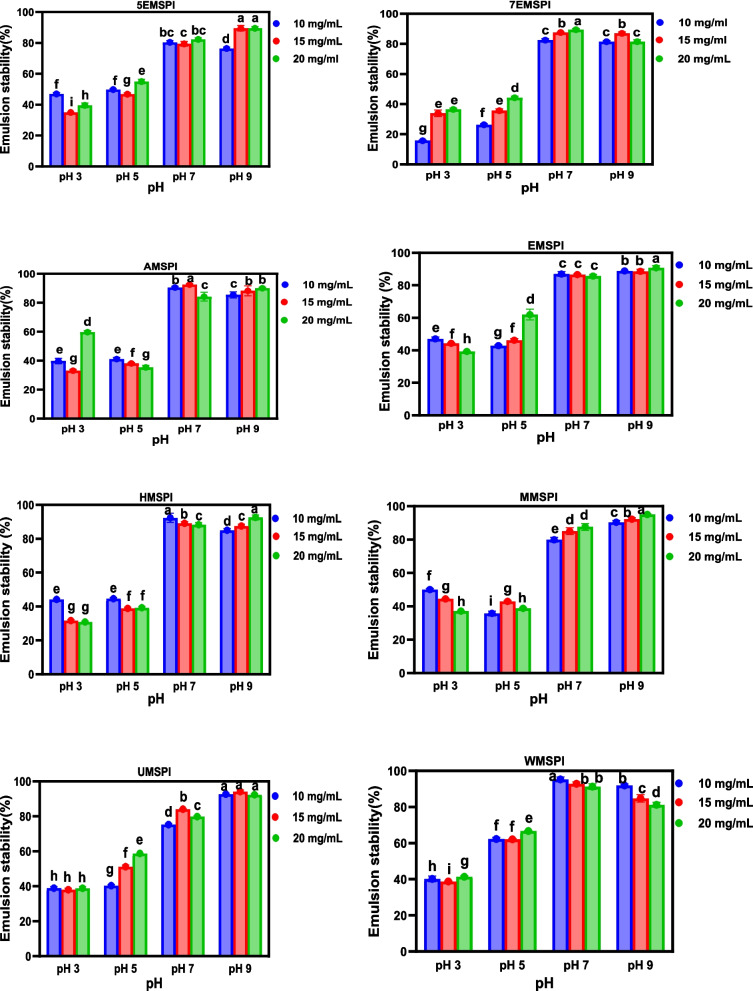
Stability of emulsions formed with protein isolates obtained from undefatted *Moringa Stenopetala* meal (UMSPI) or meal defatted with; acetone (AMSPI), 100% ethanol (EMSPI), hexane (HMSPI), methanol (MMSPI), 70% ethanol (7EMSPI), 50% ethanol (5EMSPI), and water (WMSPI). The data represent the mean values of duplicate readings with their corresponding standard deviations

As shown in Fig. 4, emulsion stability was assessed 30 min after emulsion formation and under varying protein concentrations (10, 15, and 20 mg/mL) and pH (3, 5, 7, and 9). Emulsion stability reflects the ability of protein-stabilized emulsions to resist phase separation over time (Fan et al., 2022). Stability was lowest at pH 3 and highest at pH 9. At pH 7 and 9, emulsions exhibited superior stability compared to those at pH 3 and 5, consistent with findings for hemp seed protein isolate, where stability decreased under acidic conditions (Malomo et al., 2014). Increased emulsion stability at higher pH is attributed to higher net surface charge, which contributes to increased repulsions between the interfacial protein films and hence reduced coalescence of oil droplets (Fan et al., 2022). This supports the formation of stable emulsions despite the larger droplet sizes observed at pH 7 and 9. Although smaller droplet sizes typically improve stability by increasing the surface area and reducing coalescence, the current results show the opposite. Larger droplets at higher pH levels exhibited greater stability, which aligns with the report of Kaushik et al. (2016), who noted that droplet size alone does not determine emulsion stability. Other important determinants of emulsion stability include interfacial protein charge and viscosity of the continuous phase. At pH 3, despite high solubility, the acidic environment induced structural changes in the protein that destabilized the emulsion. Acid-induced conformational changes weaken interfacial protein film, increasing susceptibility to coalescence and phase separation (Han et al., 2021). Similarly, at pH 5, which is near the pI of MSPI, emulsion stability was reduced. Close to the pI, solubility decreases, and proteins carry minimal net charge, leading to reduced electrostatic repulsion and weak interfacial films, which promote droplet coalescence and destabilization (Albano et al., 2019). These results are consistent with findings from a previous study on sesame seed protein isolates, where emulsion stability declined near the pI (Rezaei et al., 2022). Among the tested concentrations, emulsions with 20 mg/mL protein consistently showed the highest stability across most pH levels. Higher protein concentrations improved stability by increasing the availability of protein molecules at the interface, which enabled the formation of thick films that are resistant to fast coalescence and phase separation (Albano et al., 2019). WMSPI exhibited excellent stability at pH 5, suggesting that defatting the MS meal with water produced proteins with better ability to form strong and cohesive interfacial membranes near the pI. HMSPI and AMSPI also showed higher stability, particularly at pH 9, highlighting the positive effect of these solvents on protein structure with respect to emulsion stability. These results align with a previous report showing that hexane-defatted insect protein isolate achieved the highest emulsion stability (Kim et al., 2021). It is important to note that emulsion stability depends not only on solubility but also on other factors, such as phase-to-volume ratio, droplet size distribution, interfacial properties, viscosity, and density differences between phases (Mohammed et al., 2018). Each parameter plays a crucial role in determining the overall stability of protein-stabilized emulsions.

### Foaming capacity (FC) and foam stability (FS)

**Fig. 5:**
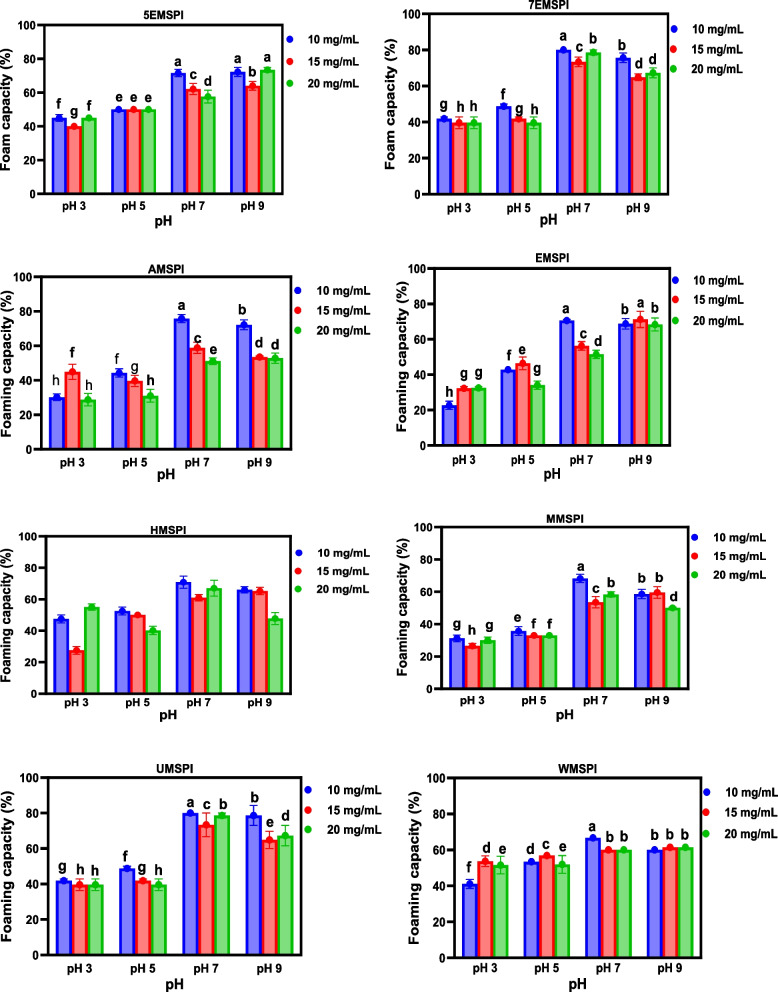
Foaming capacity of protein isolates obtained from undefatted *Moringa stenopetala* meal (UMSPI) or meal defatted with; acetone (AMSPI), 100% ethanol (EMSPI), hexane (HMSPI), methanol (MMSPI), 70% ethanol (7EMSPI), 50% ethanol (5EMSPI), and water (WMSPI). The data represent the mean values of duplicate readings with their corresponding standard deviations

Figure 5 presents the FC of MSPI at varying pH values (3, 5, 7, and 9) and concentrations (10, 15, and 20 mg/mL). FC refers to a protein's ability to generate foam when air is incorporated into a liquid, an essential property for food formulations because foam contributes to texture, appearance, and sensory appeal (Ellis & Lazidis, 2018). Several factors influence FC, including adsorption at the air–water interface, protein solubility, conformational changes, intermolecular interactions, and the formation of cohesive, viscoelastic films (Mundi & Aluko, 2012). The results indicate that FC is highly pH-dependent, with higher values (~ 80%) generally observed at pH 7 and 9 when compared to pH 3 and 5. This aligns with Chao and Aluko (2018), who reported that FC was higher at neutral or alkaline pH levels due to increased solubility, which enhanced protein flexibility and better ability to encapsulate air bubbles. The variation in FC across concentrations was also notable. In some conditions, FC increased with higher concentrations, while in others, it decreased, indicating that concentration played a nuanced role in the foam formation. The low FC at pH 3 and 5 can be attributed to the more compact protein structure, as revealed by the intrinsic fluorescence data (Fig. 1). The more compact structure would have limited the unfolding capacity needed for effective encapsulation of air bubbles. These findings emphasize that high solubility alone does not always lead to improved foaming properties.

The increased FC at pH 7, despite some aggregation, supports the idea that partial unfolding and aggregation can enhance foam formation by improving interfacial properties. This observation aligns with El Nasri and El Tinay (2007), who reported higher FC due to increased protein aggregation. Similarly, Benelhadj et al. (2016) demonstrated that raising pH enhanced the FC of algae protein isolates, suggesting that factors beyond solubility contribute to foam formation. At pH 9, the FC was notably high, likely due to improved protein solubility and conformational flexibility in alkaline conditions. This observation is further supported by the increased unordered structure at pH 7 and 9 for most samples, as revealed by the lower intrinsic fluorescence intensity when compared to pH 3 and 5. Zhan et al. (2022) also found that proteins with flexible, random coil structures were more effective in rearranging at the interface during emulsions and foam formation than highly ordered proteins, which do not unfold as easily to interact with the surrounding medium. Together, these findings suggest that enhanced conformational flexibility and protein structure at higher pH levels play a crucial role in promoting FS and FC, beyond the effects of solubility alone. Among the protein isolates tested, UMSPI showed the highest FC at pH 7 and 9. Solvent type also affected the foaming properties: 7EMSPI displayed superior FC under these conditions, indicating its potential for improving foaming performance in food systems. In contrast, WMSPI had the lowest FC at both pH 7 and 9, especially at the 10 mg/mL protein concentration. However, at pH 3 and 5, both HMSPI and WMSPI exhibited higher FC, suggesting specific effects of solvent defatting on protein conformation. These findings highlight the potential applications of MSPI in food products that require strong foaming properties, such as whipped toppings and foamy beverages.

**Fig. 6:**
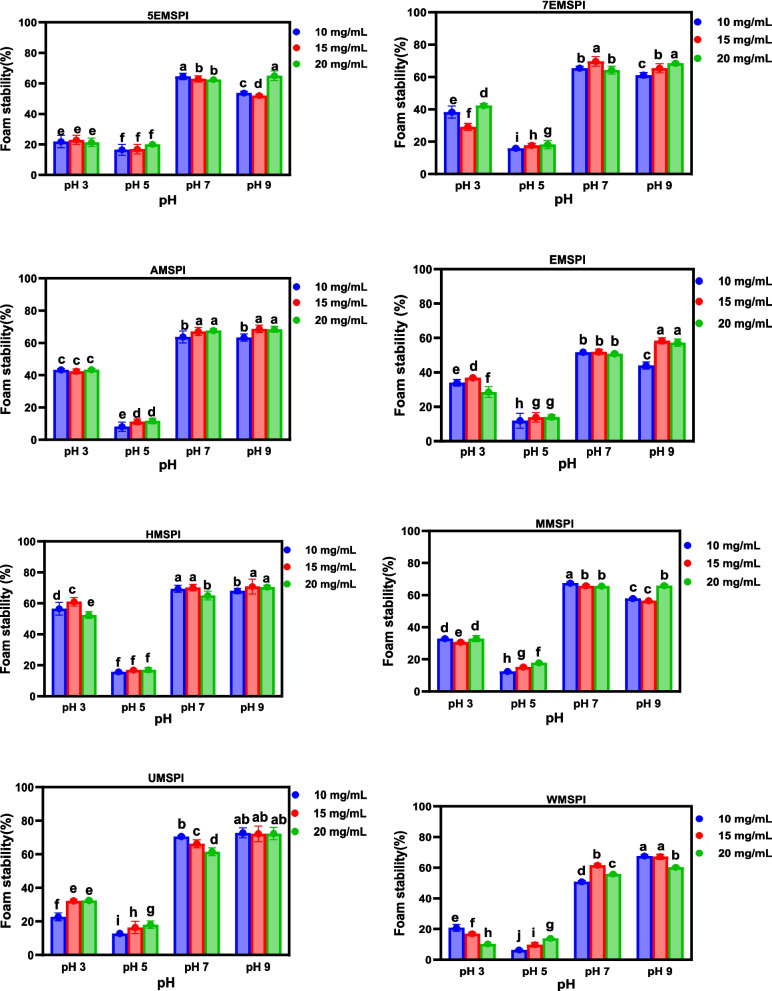
Stability of foams produced by protein isolates obtained from undefatted *Moringa stenopetala* meal (UMSPI) or meal defatted with; acetone (AMSPI), 100% ethanol (EMSPI), hexane (HMSPI), methanol (MMSPI), 70% ethanol (7EMSPI), 50% ethanol (5EMSPI), and water (WMSPI). The data represent the mean values of duplicate readings with their corresponding standard deviations

Figure 6 presents FS results for MSPI across different pH levels and concentrations. FS reflects the persistence of foam over time, a crucial property in food formulations that rely on a stable air–water interface. As with FC, the FS was influenced by pH, concentration, and solvent type. The results show higher FS at pH 3 than at pH 5. The results agree with findings from previous research, where pea protein concentrates exhibited better FS at pH 3 than at pH 5 (Asen & Aluko, 2022). Similarly, Barac et al. (2015) reported that both native and denatured legume proteins displayed enhanced FS under neutral and alkaline conditions. In this study, FS increased at pH 7 and 9, mirroring the trend observed for FC. This suggests that MSPI performs optimally in both foam formation and stability at neutral to alkaline pH levels, making it suitable for applications in whipped toppings, beverages, and desserts. The increased FS at pH 7 and 9 could be attributed to higher levels of electrostatic charges that reduced interactions between the foam particles to prevent foam collapse. Notably, 5EMSPI and WMSPI exhibited the lowest FS at pH 3 and 5, while EMSPI showed poor FS at pH 7 and 9. These differences highlight the importance of the type of solvent used during lipid removal on functionality of the isolated protein. The interactions between protein structure, solubility, concentration, and solvent type play critical roles in determining both FC and FS. These findings demonstrate that MSPI has strong potential as a functional ingredient in food systems that require foaming properties at neutral and alkaline pH environments. Understanding the interplay between pH, concentration, and defatting methods can help optimize MSPI's performance in various food formulations.

## Conclusions

This study examined how solvent extraction of MS seed meal affects the physicochemical and functional properties of the isolated MSPIs. The results indicate that pH 3 provided an environment for the highest solubility, making it suitable for applications that require good dispersion, such as protein-enriched beverages. However, the acidic nature of pH 3 may limit its applicability in certain formulations, such as emulsions due to reduced stability. On the other hand, pH 7 and pH 9 are more appropriate for emulsions and foams, as these conditions promote stability and air incorporation. The results also showed that the UMSPI retained superior functionality across multiple parameters, suggesting that lack of defatting may have preserved the native protein structure during processing, which enhanced its overall performance. AMSPI from acetone-defatted meal was the most balanced with respect to protein structural integrity and functionality across different tests. AMSPI demonstrated high solubility, good emulsifying properties, reduced heat coagulability, and strong foaming performance across various pH levels. When comparing different ethanol concentrations for MS meal defatting, 70% ethanol was the most effective in enhancing protein functionality of the extracted proteins. 7EMSPI offers a well-rounded combination of high solubility, and protein digestibility. This solvent may have contributed to maintaining a structure that enhanced protein functionality better than both 5EMSPI and EMSPI samples, making the 7EMSPI an optimal choice for food applications. These findings emphasize the importance of selecting appropriate solvent treatment of the MS meal to enhance the performance of MSPI in various food and nutraceutical formulations.

## Data Availability

Data will be available upon request.
